# A RCT of a Transdiagnostic Internet-Delivered Treatment for Three Anxiety Disorders: Examination of Support Roles and Disorder-Specific Outcomes

**DOI:** 10.1371/journal.pone.0028079

**Published:** 2011-11-23

**Authors:** Luke Johnston, Nickolai Titov, Gavin Andrews, Jay Spence, Blake F. Dear

**Affiliations:** 1 CRUfAD, School of Psychiatry, University of New South Wales at St Vincent's Hospital, Sydney, Australia; 2 eCentreClinic, Department of Psychology, Centre for Emotional Health, Macquarie University, Sydney, Australia; Research and Development Corporation, United States of America

## Abstract

**Background:**

Anxiety disorders share common vulnerabilities and symptoms. Disorder-specific treatment is efficacious, but few access evidence-based care. Administering transdiagnostic cognitive-behavioral therapy via the internet (iCBT) may increase access to evidence-based treatment, with a recent randomized controlled trial (RCT) providing preliminary support for this approach. This study extends those findings and aims to answer three questions: Is a transdiagnostic iCBT program for anxiety disorders efficacious and acceptable? Does it result in change for specific disorders? Can good clinical outcomes be obtained when guidance is provided via a Coach rather than a Clinician?

**Method:**

RCT (N = 131) comparing three groups: Clinician-supported (CL) vs. Coach-supported (CO) vs. waitlist control (Control). Individuals met DSM-IV criteria for a principal diagnosis of generalized anxiety disorder (GAD), social phobia (SP) or panic disorder with or without agoraphobia (Pan/Ag). Treatment consisted of an 8-lesson/10 week iCBT program with weekly contact from a Clinician or Coach, and follow-up at 3-months post-treatment.

**Results:**

Outcomes for the pooled treatment groups (CL+CO) were superior to the Control group on measures of anxiety, depression and disability, were associated with medium to large effect sizes (Cohen's *d* = .76 – 1.44) (response rate = 89–100%), and were maintained at follow-up. Significant reductions were found on disorder-specific outcomes for each of the target diagnoses, and were associated with large effect sizes. CO participants achieved similar outcomes to CL participants at post-treatment, yet had significantly lower symptom severity scores on general anxiety, panic-disorder, depression and disability at follow-up (*d* = .45 – .46). Seventy-four percent of CO and 76% of CL participants completed the program. Less than 70 minutes of Clinician or Coach time was required per participant during the program.

**Discussion:**

This transdiagnostic iCBT course for anxiety appears to be efficacious, associated with significant change for three target disorders, and is efficacious when guided by either a Clinician or Coach.

**Trial Registration:**

Australian New Zealand Clinical Trials Registry ACTRN12610000242022

## Introduction

The anxiety disorders frequently co-occur and share similar vulnerability factors and symptoms [1]. Disorder-specific treatment protocols for anxiety are effective [2], however, less than 40% of people with anxiety access mental health services [3]. There is growing interest in improving access and availability of treatment by integrating low-intensity treatments, aimed at balancing minimal intervention to maximum clinical gain, into routine care for common mental disorders such as anxiety and depression [4], [5]. Two innovative approaches that have the potential to improve the proportion treated are Internet-delivered cognitive behavioral therapy (iCBT) and transdiagnostic treatments.

iCBT programs teach the techniques of CBT in a highly structured format and involve the delivery of online lessons with remote support from a clinician. Meta-analyses of iCBT and computerized CBT for anxiety disorders and depression indicate that these treatments produce superior effect sizes over control conditions and are comparable to face-to-face treatments [6], [7], [8]. Moreover, three studies compared clinical and non-clinical support roles for guided iCBT and revealed no difference in efficacy [9], [10], [11].

The second innovative approach is the use of transdiagnostic protocols, also described as unified [12] or broad spectrum [13] treatments. Transdiagnostic protocols can be considered as those that apply treatment principles that are common to similar mental disorders without being tailored to, or requiring knowledge of, a specific diagnosis to be effective [14], [15], [16]. While these protocols have been created for the treatment of similar disorders, an unresolved issue in this area concerns which disorders are suitable for transdiagnostic treatment, particularly regarding PTSD and OCD relative to other anxiety disorders [17]. Despite this, meta-analyses of a relatively small number of studies indicate that face-to-face transdiagnostic treatments may result in similar outcomes on generic anxiety measures to disorder-specific treatments [16], [18].

Evidence for the effectiveness of computer-delivered transdiagnostic treatments was provided by two early studies that used computerized CBT to treat panic and phobias, and anxiety and depression, demonstrating symptom reduction from single treatment protocols [19], [20]. These programs have now been integrated within the UK Improved Access to Psychological Therapies program, stepping consumers from low to high-intensity treatments as clinically required, with completer analyses demonstrating good outcomes [4]. Additionally, preliminary support for the efficacy of an internet-delivered transdiagnostic treatment was reported in a recent RCT examining a transdiagnostic iCBT program for generalized anxiety disorder (GAD), social phobia (SP) and panic disorder (with or without agoraphobia) (Pan/Ag), demonstrating reductions in anxiety, distress and disability [21]. The lessons in this program covered core components including psychoeducation, cognitive restructuring, graded exposure, troubleshooting common treatment difficulties and relapse prevention. Additional resources covering issues such as low mood, improving sleep and communication skills, were accessed by all participants and were not prescribed. However, the sample size was not sufficient to reliably examine whether changes occurred in outcome measures for each of the three anxiety disorders.

The present study was an extension of the aforementioned study [21], using a larger sample to explore a revised version of the program, and also compared the relative benefits of Coach against Clinician guidance. This study attempted to answer three questions:

1. Is a transdiagnostic iCBT program for anxiety disorders efficacious and acceptable?
2. Does the program result in change for each specific disorder?
3. Can good clinical outcomes be obtained when support is provided by a Coach when compared with a Clinician?

## Methods

The protocol for this trial and supporting CONSORT checklist are available as supporting information; see Checklist S1 and Protocol S1.

### Design and objectives

The objectives were to determine (1) the efficacy of a transdiagnostic iCBT program for anxiety disorders, (2) the relative benefits to each of the target disorders, and (3) to compare outcomes when the program is supported by either a Clinician or Coach. The design comprised a CONSORT-R compliant RCT comparing three parallel conditions: A Clinician-assisted iCBT treatment group (CL group); a Coaching-assisted iCBT treatment group (CO group); and a waitlist deferred-treatment control group (Control).

### Hypotheses

The three hypotheses were: 1) The pooled CL and CO group (CL+CO) participants would show significant improvement on general and disorder-specific measures of anxiety, and measures of depression and disability after treatment, relative to Control participants, and would rate the treatment as acceptable; 2) the pooled CL+CO participants would show significant improvement on disorder-specific measures of anxiety over time, and; 3) participants in the CO group would achieve at similar outcomes to the CL group across all measures and time points.

### Ethics

The study was approved by the Human Research Ethics Committee (HREC) of St Vincent's Hospital (Sydney, Australia) and the HREC of the University of New South Wales (Sydney, Australia). All participants provided written informed consent. The trial was registered as ACTRN12610000242022.

### Participants

**Figure 1:**
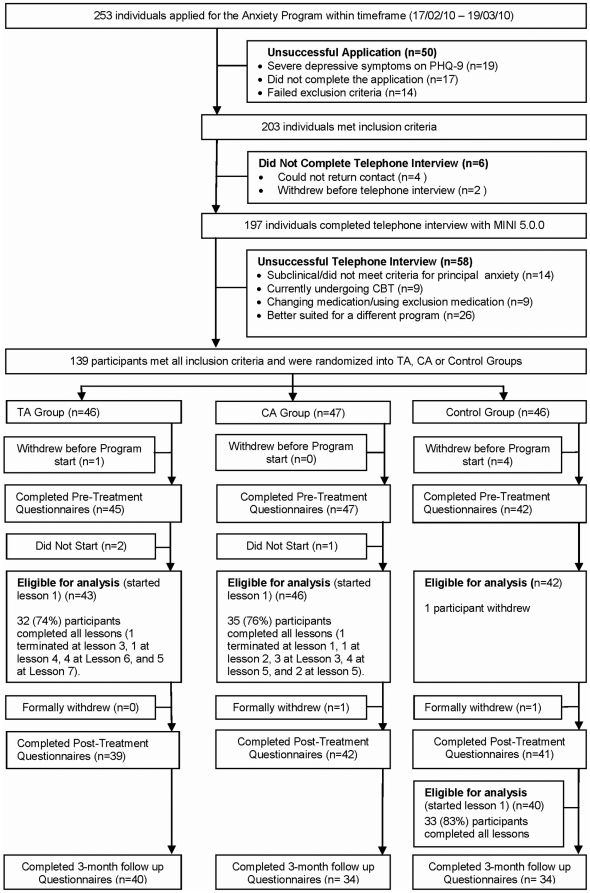
CONSORT-R participant flow chart. PHQ-9, Patient Health Questionnaire – 9 Item; MINI 5.0.0, Mini International Neuropsychiatric Interview.

Potential participants consisted of individuals who had previously expressed interest in treatment via the online programs available on a research website, or from visitors to the website. Applicants applied online to the research website where they read details about the study. Details of participant flow are in Figure 1. During the 4 weeks of recruitment in early 2010, 253 individuals applied and 139 met the following inclusion criteria: (i) resident of Australia; (ii) at least 18 years of age; (iii) access to a computer, the Internet, and use of a printer; (iv) not currently participating in CBT; (v) not using illicit drugs or consuming more than three standard drinks a day; (vi) not currently experiencing a psychotic mental illness or severe symptoms of depression (defined as a total score >22 or responding >2 to Question 9 (suicidal ideation) on the Patient Health Questionnaire - 9 Item [22]; (vii) if taking medication (people taking benzodiazepines were excluded), had been taking the same dose for at least 1 month and did not intend to change that dose during the course of the program; and (viii) met DSM-IV [23] diagnostic criteria for a principal diagnosis (defined as the disorder the participant nominated as most troubling) of GAD, SP, or Pan/Ag. Applicants who did not meet these criteria were informed via an on-screen message and were sent an email thanking them for their application and encouraging them to discuss their symptoms with their physician. Participants who met the inclusion criteria then completed a 25-item questionnaire enquiring about demographic details and treatment history (see Table 1).

**Table 1:** Demographic characteristics of the Coach assisted, Clinician assisted and Control groups.

	*CO Group*		*CL Group*		*Control Group*		*Total*		Statistical significance
Variable	*n*	%	*n*	%	*n*	%	*n*	%	
**Gender**									
Male	15	34.9	23	50.0	16	38.1	54	41.2	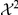 (2,N = 131) = 2.35, p = 0.31
Female	28	65.1	23	50.0	26	61.9	77	58.8	
**Age**									
Mean	38.63 (11.56)	-	43.74 (13.36)	-	42.36 (13.20)	-	41.62 (12.83)	-	F_2,128_ = 1.89, p = 0.16
Range	19-59	-	20-69	-	21-79	-	19–79	-	
**Marital Status**									
Single/Never Married	13	30.2	12	26.1	14	33.3	39	29.8	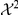 (4, N = 131) = 5.29, p = 0.26
Married/De Facto	26	58.1	20	43.5	20	47.6	65	49.6	
Separated/Divorced	5	11.6	14	30.4	8	19.0	27	20.6	
**Education**									
High school	10	23.3	8	17.4	7	16.7	25	19.1	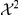 (6, N = 131) = 5.48, p = 0.48
Tertiary	29	67.4	30	65.2	25	59.5	84	64.1	
Other Certificate	4	9.3	7	15.2	10	23.8	21	16.0	
None	0	0.0	1	2.2	0	0	1	.8	
**Employment Status**									
Part time/student	19	44.2	14	30.4	19	36.5	52	39.7	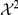 (4,N = 131) = 2.63, p = 0.62
Full time	18	41.9	23	50.0	17	40.5	58	44.3	
Unemployed, retired or disabled	6	14.0	9	19.6	6	14.3	21	16.0	
**Previously Mental Health Treatment**	29	67.4	32	69.6	31	73.8	92	70.2	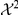 (2, N = 131) = 0.43, p = 0.81
**Taking Medication**	11	25.6	18	39.1	9	21.4	38	29.0	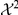 (2, N = 131) = 3.71, p = 0.16
									
**Contact During Program**	**Mean**	**SD**	**Mean**	**SD**	**Mean**	**SD**			
Number of phone calls	7.56	1.19	7.54	2.43	-	-	-	-	t_87_ = .32, p = 0.98
Number of manual written contacts	8.88	4.38	8.83	3.19	-	-	-	-	t_87_ = .87, p = 0.94
Number of automated written contacts	19.37	1.75	20.43	3.50	-	-	-	-	t_87_ = .−1.79, p = 0.08
Total contact time (min)	69.09	30.75	69.59	32.29	-	-	-	-	t_87_ = −.07, p = 0.94

Applicants who passed the screening phase were telephoned and administered a diagnostic interview using the Mini International Neuropsychiatric Interview Version 5.0.0 (MINI) [24] to determine whether they met DSM-IV criteria [23] for GAD, SP or Pan/Ag. Applicants who satisfied all criteria and completed a consent form were included in the study.

### Interventions

Both treatment groups received access to the *Anxiety Program* [21]. The intervention used in the present study employed a version with the following changes: i) information about cognitive skills were presented in the second rather than third lesson; ii) two new lessons were added to address core beliefs, beliefs about anxiety, and assertive communication and interpersonal boundaries; iii) the duration of the program was increased from eight to ten weeks. The enhanced Anxiety Program comprised the following components: Eight online lessons; a summary/homework assignment for each lesson; weekly telephone or email/asynchronous messaging contact with the Clinician or Coach, and regular automated reminder and notification emails. All participants also had access to additional written resources that included guidelines about managing low mood, improving sleep, and answers to frequently asked questions about the application of skills described in the lessons and summaries, although these were not prescribed as per other treatment protocols [25]. Participants were also provided with access to de-identified vignettes written by participants in previous iCBT programs covering topics relevant to each of the eight lessons. The content of each lesson is described in Table 2.

**Table 2:** Content of the Anxiety Program.

*Lesson*	*Primary content/theme*	*Secondary content/theme*	*De-identified vignettes*
1	Education about the prevalence, symptoms and treatment of anxiety including an explanation of the functional relationship between symptoms	Examples describing symptomsNormalising difficulties during recovery	*Examples of symptoms and their impact, and outlook on treatment*
2	Basic principles of cognitive therapy, including strategies for monitoring and challenging thoughts, and structured problem solving		*Providing examples of unhelpful thoughts and examples of challenges to thoughts*
3	Instructions about controlling physical symptoms including de-arousal strategies and scheduling activities	The importance of lifestyle factors	*Examples of using de-arousal strategies introducing lifestyle changes*
4	Education and guidelines about practicing graded exposure	Normalising difficulties with exposure and creating realistic treatment goals	*Providing examples of exposure tasks*
5	Education and guidelines about advanced cognitive skills including belief challenging	*Consolidating thought challenging tasks*	*Normalising distressing beliefs, examples of the impact of successfully challenging beliefs*
6	Education and guidelines for acting “as if” and troubleshooting common barriers to treatment		*Reporting examples of hurdles or barriers to treatment and attempts at overcoming these*
7	Education and guidelines surrounding assertive communication and interpersonal boundaries	Communication skills	*Examples of how communication styles and interpersonal boundaries contribute to anxiety, and can be managed*
8	Information about relapse prevention and constructing relapse prevention plans		*Relapse prevention plan and reviewing key skills from program*

Each Lesson began with a restatement of the key skills described in previous lessons, an introduction to skills described in the current lesson, illustrated examples about people with each of the target disorders practicing those skills, and a summary of the main points. Participants were encouraged to complete one lesson each week, to complete the recommended homework and to complete the eight lessons within 10 weeks.

### Clinician/Coach roles

Two staff conducted the study with supervision from NT. The Clinician role was fulfilled by JS who had completed specialist post-graduate training in Clinical Psychology, had 2.5 years post-clinical training experience, had previously treated participants using iCBT in two other trials [9], [21], and was employed as a Clinical Psychologist at the Anxiety Disorders Clinic, St Vincent's Hospital Sydney. The Coach role was performed by LJ, a Registered Psychologist without specialist post-graduate training, employed as a Research Assistant at the same research unit.

Clinician and Coach roles performed specific and distinct functions. Both roles required strict adherence to a pre-determined script to be followed throughout all contact with participants that specified: Reinforcing progress to date; encouraging the completion of further lessons; encouraging practice of homework tasks; normalising difficulties with practicing homework tasks; and providing direction to upcoming materials. In the event of receiving clinical questions the Coach was instructed to direct the participant to the program content or inform of upcoming materials that would address the question. The Coach was not permitted to provide clinical advice or to elaborate, expand upon or add to the existing information or skills provided in the program. The Clinician, however, was invited to provide therapy and engage the participant in more detailed discussion of the materials including how to apply the treatment, to provide further detail about the skills, assist the participant in practicing those skills, and suggest additional skills if applicable. Both Clinician and Coach received weekly supervision from an independent clinical psychologist (NT) as a matter of routine professional and ethical care. These sessions allowed discussion of clinical issues, and the opportunity for the Coach to refer participants to the Clinician in the event of any perceived deterioration in the participants' mental health status, or of any concerns about participants' wellbeing. Supervision was also provided to reinforce adherence to the script and guidelines and ensure that the Coach did not attempt ‘therapy’. Both Clinician and Coach were advised to limit weekly contact time to approximately 10 minutes per participant, unless more time was clinically indicated. Every instance of contact with each participant was recorded as was the total time that the Clinician and Coach spent per participant.

### Outcomes

A wide range of measures were used. The total number of questionnaire items was kept below 80 to reduce burden on participants. The Mini International Neuropsychiatric Interview Version 5.0.0 (MINI) [24] was used as a diagnostic measure. The Generalized Anxiety Disorder 7-Item Scale, (GAD-7) [26] and Depression Anxiety Stress Scales – 21 Item (DASS-21) [27] were used as primary outcome measures. The Penn State Worry Questionnaire (PSWQ) [28], Social Interaction Anxiety Scale and Social Phobia Scale – Short Form (SIAS-6/SPS-6) [29] and Panic Disorder Severity Scale – Self Rating (PDSS-SR) [30] were used as disorder-specific outcome measures. The Patient Health Questionnaire – 9 Item (PHQ-9) [22] and Sheehan Disability Scales (SDS) [31] were used as secondary outcome measures. Each of these measures will be discussed in detail below.

#### Mini International Neuropsychiatric Interview Version 5.0.0 (MINI) [24]

The MINI is a brief diagnostic interview developed to determine the presence of current and lifetime Axis-I disorders using DSM-IV diagnostic criteria and was chosen in favour of other diagnostic interviews to reduce participant burden. Psychometric evaluations of the MINI [32] indicate it has excellent inter-rater reliability (*k* = .88 – 1.00) and adequate concurrent validity with the Composite International Diagnostic Interview. Pre-treatment interviews were conducted by LJ and JS. Three-month follow-up interviews were conducted by LJ, JS and BD.

#### Generalized Anxiety Disorder 7-Item Scale, (GAD-7) [26]

The GAD-7 comprises seven items measuring symptoms and severity of GAD based on the DSM-IV diagnostic criteria for GAD. The GAD-7 has good internal consistency (.89) and good convergent validity with other anxiety scales [33]. Evidence indicates the GAD-7 is sensitive to GAD, social phobia, and panic disorder with increasing scores indicating greater severity of symptoms [34]. The GAD-7 is increasingly used in research and in large scale dissemination studies as a generic measure of change in anxiety symptoms [4], [35]. The internal consistency of the GAD-7 in the current study was high (Cronbach's α = .86).

#### Depression Anxiety Stress Scales – 21 Item (DASS-21) [27]

The DASS-21 is a measure of severity of symptoms for anxiety, stress, and depression, and is used to measure change in higher-order, or common symptoms across anxiety and depressive disorders. It comprises three subscales that assess features uniquely associated with depression, anxiety, and psychological distress. The 21-item short form has demonstrated excellent psychometric properties including good internal consistency and concurrent validity comparable with the original 42-item measure [36]. The internal consistency of the DASS-21 in the current study was high (Cronbach's α = .88).

#### Penn State Worry Questionnaire (PSWQ) [28]

The PSWQ consists of 16 items and is considered a valid clinical measure of worry characteristic of GAD. Early psychometric evaluations revealed the PSWQ had high internal consistency and temporal stability [28], and was able to differentiate patients with GAD from those with other anxiety disorders [37]. The internal consistency (Cronbach's α) of the PSWQ in the current study was .90.

#### Social Interaction Anxiety Scale and Social Phobia Scale – Short form (SIAS-6/SPS-6) [29]

The SIAS-6/SPS-6 is a recently developed brief measure of social anxiety (12 items) based on the items of the Social Interaction Anxiety Scale (SIAS) and the Social Phobia Scale (SPS) [38]. The measure correlated strongly and significantly with the SIAS and SPS in clinical samples at pre-treatment, post-treatment, and at 3-month follow-up (*rs* = .79 –.90), and also correlated strongly and significantly with change scores in the SIAS and SPS following treatment (*rs* = .81 –.91). Cronbach's α of the SIAS-6/SPS-6 in the current study was .92.

#### Panic Disorder Severity Scale – Self Rating (PDSS-SR) [30]

The PDSS-SR is a seven-item measure of panic disorder severity. Psychometric evaluations suggest it has excellent psychometric properties including high internal consistency (Cronbach's α = .92), good test-retest reliability (*r* = .81), and sensitivity to change [30]. Cronbach's α of the PDSS-SR in the current study was high (.92).

#### Patient Health Questionnaire – 9 Item (PHQ-9) [22]

The PHQ-9 is a nine-item measure of the symptoms and severity of major depressive disorder based on the DSM-IV criteria for depression. A total score of 10 on the PHQ-9 has also been identified as an important threshold for identifying DSM-IV congruent depression with increasing scores indicating greater symptom severity [22]. Psychometric studies indicate the internal consistency is high (.86 – .89) [22] and the measure is sensitive to change [33]. The internal consistency of the PHQ-9 in the current study was high (Cronbach's α = .84).

#### Sheehan Disability Scales (SDS) [31]

The SDS comprises three items measuring impairment in psychosocial functioning with high internal consistency (α = .89) [39]. The internal consistency (Cronbach's α) of the SDS in the current study was .83.

With the exception of the SIAS-6/SPS-6, which is still under evaluation, all of these measures are considered reliable, valid, and appropriate for clinical research purposes. Moreover, recent research indicates that online administration of questionnaires results in acceptable reliability of responses with emerging evidence for equivalence between paper-and-pencil versions of self-report questionnaires and online administration [40], [41], [42], [43]. At post-treatment, all participants also completed a 7-item treatment satisfaction questionnaire based on the Credibility/Expectancy Questionnaire (CEQ) [44]. Additional questions enquiring about treatment satisfaction with the transdiagnostic treatment protocol were administered to participants in the treatment groups at follow-up. All questionnaires were administered via the Internet.

### Timepoints

All participants were asked to complete the questionnaire outcome measures (GAD-7, DASS-21, PSWQ, SIAS-6/SPS-6, PDSS-SR, PHQ-9 and SDS) at pre-treatment, post-treatment, and at 3-month follow-up. Control group participants began treatment immediately after the CL and CO post-treatment time point, so the 3-month follow up for the CL and CO groups coincided with the post-treatment time point for the Control group.

### Sample size and randomization

Power calculations indicated that a sample size of 36 participants in each group was sufficient to detect an effect size (ES) difference of 0.6 between the treatment groups and the Control group, with alpha at .05 and power of 80%, which was the minimum expected based on similar studies [9], [45], [46]. The study was not powered to detect small differences between the treatment groups.

One-hundred and thirty-nine applicants met all inclusion criteria and were randomized via a true randomization process (www.random.org), generated by an independent person, to either CL, CO or Control groups. The allocation sequence preceded pre-treatment diagnostic interviews and was concealed from LJ and JS. Researchers completing 3-month follow-up interviews were not blind to group allocation. Dependence on self-report measures precluded blinding.

### Statistical analyses

#### Analysis of primary, disorder-specific and secondary outcome measures

Baseline between-group differences in demographic data and pre-treatment measures were analysed with *one-way ANOVAs* and *chi*-square tests. To determine whether the transdiagnostic iCBT program was efficacious, scores from the CL and CO groups were pooled to create a single *CL+CO* group. To explore the relative clinical outcomes of each type of support, CL and CO data were analysed separately.

All post-treatment and 3-month follow-up analyses involved an intention-to-treat (ITT) design and missing data was addressed by carrying forward the first available data (baseline-observation-carried-forward; BOCF). Between-group changes in questionnaire scores were analysed using *univariate ANCOVAs*, assigning pre-treatment scores as the covariate. This approach is recommended as a robust and reliable statistical strategy for analysing the results of RCTs [47], [48]. Independent-samples *t*-tests were used to assess between-groups differences in the number and duration of contacts with participants. Within-group changes in questionnaires were analysed using paired-samples *t*-tests. Effect sizes (Cohen's *d*) were calculated for within- and between-group changes, based on the pooled standard deviation.

#### Clinical significance

Two criteria of clinical significance were employed. Pre-treatment, post-treatment and 3-month follow up GAD-7 scores were compared with clinical cut-offs to provide an index of *remission.* This was defined as the proportion of participants who initially scored at or above, and subsequently scored below the cut-offs of a GAD-7 total score ≥8 [34]. An estimate of *recovery* was made by identifying the proportion of participants in each group who scored above the aforementioned pre-treatment threshold and subsequently demonstrated a significant reduction in their symptoms (defined here, as a reduction of 50% of pre-treatment GAD-7), as described in recent dissemination studies [4]. Secondly, changes in prevalence of principal and additional disorders of anxiety in the two treatment groups were calculated on the results of the diagnostic interviews conducted at pre-treatment and 3-month follow-up and were analysed with *chi*-square tests. All quantitative analyses were performed in PASW version 18.0 (SPSS, Inc., Chicago, IL).

#### Control group results

As a preliminary test of the reliability of outcome associated with the Coach condition, data from the Control group, following their treatment, is reported.

## Results

### Participant flow

Two hundred and fifty-three individuals expressed interest in the study, and 139 met the eligibility criteria and were randomized to one of the three groups. One CO group and four Control group participants withdrew before beginning the program. Additionally, two CO group and one CL group participant withdrew before beginning treatment, which resulted in 43 CO, 46 CL and 42 Control group participants eligible for analysis (see Figure 1).

### Baseline data

Table 1 shows the demographic characteristics of each group and the overall sample. There were no significant between-group differences in gender, marital status, education, employment, previous discussions of symptoms with a health professional, use of medication (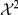 range(2–6, N = 131) = 0.76 – 8.60, *p* range = .15 – .69), age or treatment expectancy (*F_2, 128_* range = .62 – 1.89, *p* range = .15 – .51).

Principal and additional diagnoses are displayed in Table 3. Twenty-nine of 43 (67%) CO, 35/46 (76%) CL and 28/42 (67%) Control participants had a co-morbid anxiety or depressive disorder (70% of the overall sample). At pre-treatment, GAD was the most common principal disorder followed by SP and Pan/Ag. There were no statistically significant differences between groups in the prevalence of each principal diagnosis, or the presence of additional diagnoses (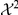
(2–4, N = 131) range = 1.09 – 1.17, *p* range = .56 – .90).

**Table 3:** Frequency of principal diagnoses and comorbidity for Treatment and Control groups at pre-treatment, and 3-month follow up for CO and CL groups.

	Pre-treatment								3-month follow-up					
	CO Group		CL Group		Control Group		Total		CO Group		CL Group		Total	
	N	%	N	%	N	%	N	%	N	%	N	%	N	%
**Principal diagnosis**														
GAD	18	41.9	21	45.7	20	47.6	59	45.0	7	16.3	9	19.6	16	18.0
SP	14	32.6	16	34.8	15	35.7	45	34.4	5	11.6	11	23.9	16	18.0
Pan/Ag	11	25.6	9	19.6	7	25.9	27	20.6	5	11.6	6	13.0	11	12.8
**Comorbid condition**														
None	14	32.7	11	23.9	14	33.3	39	29.8	35	81.4	26	56.5	61	68.5
Anxiety only	13	30.2	14	30.4	11	26.2	38	29.0	3	7.0	11	23.9	14	15.7
Affective only	3	7.0	7	15.2	2	4.8	12	9.2	1	2.3	1	2.2	2	2.2
Anxiety and affective only	13	30.2	14	30.4	15	35.7	42	32.1	4	9.3	8	17.4	12	13.5
**Number of additional diagnoses**														
0	14	32.6	11	23.9	14	33.3	39	29.8	35	81.4	26	56.5	61	68.5
1	13	30.2	17	37.0	9	21.4	39	29.8	4	9.3	7	15.2	11	12.4
2	8	18.6	11	23.9	13	31.0	32	24.4	1	2.4	10	21.7	11	12.4
3+	8	18.6	7	15.2	6	14.3	21	16.0	3	7	3	6.5	6	6.7

*Note:* Intention-to-treat model was employed with pre-treatment diagnoses being carried forward if follow-up data was not available. Diagnostic interviews were not repeated with Control group as they had begun treatment. Abbreviations: GAD, Generalised Anxiety Disorder; SP, social phobia; Pan/Ag, panic disorder with or without agoraphobia; CO: Coach-assisted; CL: Clinician-assisted.

Table 4 shows the pre-treatment scores for the pooled CL+CO group and for the Control group, and Table 5 shows the pre-treatment scores for the CO and CL groups separately, on primary, disorder-specific and secondary outcome measures. There was a trend towards significance between groups in pre-treatment scores on the DASS-21 (*F_2, 128_* = 2.83, *p* = .06), and no significant differences between CO, CL and Control groups in pre-treatment scores on the GAD-7, PSWQ, PDSS-SR, SIAS-6/SPS-6, PHQ-9 or SDS (*F_2, 128_* range = .07 – .79, *p* range = .45 – .94).

**Table 4:** Means, standard deviations and effect sizes (Cohen's d) for pooled CL+CO and Control groups on all outcome measures at pre-treatment, post-treatment and 3-month follow-up time points.

Measure and group	Pre-treatment Mean	Post-treatment Mean	Follow-up Mean	Within-group effect size		Between-group effect size
				Pre- to post-treatment	Pre-treatment to follow-up	Post-treatment
**GAD-7**						
CL+CO (n = 89)	11.71 (4.34)	6.17 (4.38)	6.61 (5.54)	1.28 (.38 – 2.19)	1.03 (.13 – 2.18)	1.44 (.05 – 2.31)
Control (n = 42)	12.50 (4.80)	11.79 (4.60)	5.70 (3.53)	0.15 (−1.30 – 1.54)	1.63 (.18 – 2.70)	-
**DASS-21**						
CL+CO (n = 89)	50.70 (21.75)	28.67 (21.71)	27.35 (25.14)	1.02 (−3.50 – 5.53)	1.00 (−3.52 – 6.22)	.94 (−5.24 – 5.45)
Control (n = 42)	52.57 (20.86)	48.48 (20.41)	24.25 (16.54)	0.20 (−6.11 – 6.37)	1.52 (−4.79 – 6.52)	-
**PSWQ**						
CL+CO (n = 89)	63.63 (11.01)	52.07 (10.70)	52.06 (13.37)	1.07 (−1.22 – 3.29)	.95 (−1.34 – 3.73)	.83 (−3.02 – 3.06)
Control (n = 42)	61.29 (12.66)	61.50 (12.74)	50.05 (11.23)	0.02 (−3.85 – 3.84)	0.95 (−2.85 – 4.35)	-
**SIAS-6/SPS-6**						
CL+CO (n = 89)	20.31 (11.45)	12.56 (9.03)	13.26 (10.53)	.76 (−1.62 – 2.63)	0.64 (−1.73 – 2.83)	.89 (−3.30 – 2.76)
Control (n = 42)	22.17 (13.59)	22.05 (13.83)	14.53 (11.10)	0.01 (−4.10 – 4.19)	0.62 (−3.49 – 3.98)	-
**PDSS-SR**						
CL+CO (n = 89)	10.20 (6.89)	5.71 (5.80)	5.97 (7.31)	.71 (−.72 – 1.91)	.60 (−.83 – 2.12)	.81 (−1.11 – 2.01)
Control (n = 42)	10.74 (6.44)	10.50 (6.35)	5.58 (5.03)	0.04 (−1.91 – 1.96)	0.90 (−1.05 – 2.46)	-
**PHQ-9**						
CL+CO (n = 89)	11.46 (5.57)	6.88 (5.21)	6.76 (6.00)	.85 (−.30 – 1.94)	.82 (−.34 – 2.06)	.85 (−.75 – 1.93)
Control (n = 42)	11.71 (6.31)	11.29 (5.28)	11.29 (5.28)	0.07 (−1.84 – 1.67)	0.88 (−1.03 – 2.42)	-
**SDS**						
CL+CO (n = 89)	17.17 (7.06)	10.15 (7.54)	9.27 (8.82)	.97 (−.50 – 2.53)	.99 (−.47 – 2.83)	.76 (−1.58 –2.33)
Control (n = 42)	16.43 (7.74)	15.88 (7.75)	9.40 (7.71)	0.07 (−2.27 – 2.42)	0.92 (−1.42 – 3.25)	-

*Note.* The standard deviations of the means and the confidence intervals of effect sizes are shown in parentheses. Intention-to-treat model was employed with pre-treatment scores being carried forward if post-treatment or follow-up data was not available Abbreviations: GAD-7: Generalized Anxiety Disorder 7-Item; DASS-21: Depression Anxiety Stress Scales-21 item; PSWQ: Penn State Worry Questionnaire; SIAS-6/SPS-6: Social Interaction Scale and Social Phobia Scale Short Form; PDSS-SR: Panic Disorder Severity Scale – Self Rating; PHQ-9: Patient Health Questionnaire-9 item; SDS: Sheehan Disability Scale. CO: Coach-assisted; CL Clinician-assisted.

**Table 5:** Means, standard deviations and effect sizes (Cohen's d) for CO and CL groups on all outcome measures at pre-treatment, post-treatment and 3-month follow-up timepoints.

Measure and group	Pre-treatment Mean	Post-treatment Mean	Follow-up Mean	Within group effect size		Between group effect size	
				Pre- to post-treatment	Pre-treatment to follow-up	Post-treatment	Follow-up
**GAD-7**							
CO (n = 43)	11.28 (5.18)	6.16 (4.59)	5.37 (4.98)	1.06 (−0.49 – 1.06)	1.18 (−0.37 – 2.67)	0.27 (−1.38 – 1.64)	0.46 (−1.45 – 1.95)
CL (n = 46)	11.63 (5.96)	7.54 (5.70)	8.07 (6.61)	0.71 (−1.01 – 2.36)	0.57 (−1.15 – 2.48)	-	-
**DASS-21**							
CO (n = 43)	45.30 (19.54)	22.05 (16.90)	21.16 (22.27)	1.29 (−4.55 – 6.34)	1.17 (−4.67 – 7.82)	0.62 (−6.30 – 5.67)	0.49 (−7.16 – 7.15)
CL (n = 46)	55.74 (22.69)	34.87 (23.95)	33.13 (26.49)	0.90 (−5.65 – 7.83)	0.93 (−5.63 – 8.58)	-	-
**PSWQ**							
CO (n = 43)	62.81 (11.35)	50.28 (10.34)	49.86 (12.00)	1.17 (−2.22 – 4.26)	1.12 (−2.27 – 4.71)	0.52 (−2.62 – 3.65)	0.33 (−3.82 – 3.96)
CL (n = 46)	64.39 (10.75)	53.74 (10.86)	54.19 (14.37)	0.81 (−2.30 – 3.95)	0.81 (−2.39 – 4.97)	-	-
**SIAS-6/SPS-6**							
CO (n = 43)	19.95 (12.84)	10.95 (8.98)	11.65 (9.64)	0.82 (−3.02 – 3.51)	0.74 (−3.10 – 3.62)	0.35 (−2.22 – 3.04)	0.30 (−2.94 – 3.18)
CL (n = 46)	20.65 (10.12)	14.07 (8.90)	14.76 (11.20)	0.70 (−2/23 – 3.27)	0.56 (−2.37 – 3.79)	-	-
**PDSS-SR**							
CO (n = 43)	9.72 (6.89)	4.95 (4.99)	4.30 (6.68)	0.80 (−1.26 – 2.29)	0.81 (−1.25 – 2.80)	0.26 (−1.61 – 1.75)	0.45 (−1.74 – 2.45)
CL (n = 46)	10.65 (6.93)	6.41 (6.44)	7.52 (7.59)	0.67 (−1.19 – 2.53)	0.45 (−1.40 – 2.64)	-	-
**PHQ-9**							
CO (n = 43)	11.28 (5.18)	6.16 (4.59)	5.37 (4.98)	1.06 (−0.49 – 1.06)	1.18 (−0.37 – 2.67)	0.27 (−1.38 – 1.64)	0.46 (−1.45 – 1.95)
CL (n = 46)	11.63 (5.96)	7.54 (5.70)	8.07 (6.61)	0.71 (−1.01 – 2.36)	0.57 (−1.15 – 2.48)		-
**SDS**							
CO (n = 43)	16.23 (6.37)	8.35 (6.72)	6.84 (7.56)	1.22 (−0.69 – 3.23)	1.36 (−0.54 – 3.62)	0.48 (−1.81 – 2.51)	0.56 (−2.15 – 2.84)
CL (n = 46)	18.04 (7.62)	11.83 (7.93)	11.54 (9.37)	0.81 (−1.39 – 3.10)	0.77 (−1.43 – 3.48)	-	-

*Note.* The standard deviations of the means and the confidence intervals of effect sizes are shown in parentheses. Intention-to-treat model was employed with pre-treatment scores being carried forward if post-treatment or follow-up data was not available Abbreviations: GAD-7: Generalized Anxiety Disorder 7-Item; DASS-21: Depression Anxiety Stress Scales-21 item; PSWQ: Penn State Worry Questionnaire; SIAS-6/SPS-6: Social Interaction Scale and Social Phobia Scale Short Form; PDSS-SR: Panic Disorder Severity Scale – Self Rating; PHQ-9: Patient Health Questionnaire-9 item; SDS: Sheehan Disability Scale. CO: Coach assisted; CL Clinician assisted.

### Adherence and attrition

Thirty-two of 43 (74%) CO and 35/46 CL (76%) group participants completed all eight lessons within the 10-week program. A further four (9%) CO participants completed the remaining lesson within seven days of the Program ending, but no CL participants completed within that time frame. There was no difference (*t_87_* = 1.10, *p* = .27) in the mean number of lessons completed by CO group (7.57; SD = 0.99) and CL group participants (7.09; SD = 1.81). Post-treatment data was collected from 39/43 (90%) CO, 41/46 (89%) CL, and from 42/42 (100%) Control group participants. Three month follow-up data was provided by 40/43 (93%) CO and 34/46 (74%) CL group participants.

### Is a transdiagnostic iCBT program for anxiety disorders efficacious?

Univariate ANCOVAs, controlling for pre-treatment scores, on post-treatment primary, disorder-specific and secondary outcomes outcome measures (Table 4) revealed significant differences between CL+CO and Control groups on the GAD-7, DASS-21, PSWQ, SIAS-6/SPS-6, PDSS-SR, PHQ-9, and SDS (*F_2, 130_* = 28.28 – 53.68, *p*<.000). Paired samples *t*-tests revealed no significant difference between post-treatment and 3-month follow up scores for the CL+CO group (*t_88_* = −1.15 – 2.13, *p* = .13 – 99).

Between- and within-group effect sizes on primary measures are included on Table 4. Large between-group effect sizes were achieved by the CL+CO group relative to the Control group on the GAD-7, DASS-21, PSWQ, SIAS-6/SPS-6, PDSS-SR and PHQ-9, (*d* = .81 – 1.44) and a moderate effect size was found on the SDS (*d* = .76). Large within group effect sizes were achieved by the CL+CO group at post-treatment on the GAD-7, DASS-21, PSWQ, PHQ-9 and SDS (*d* = .85 – 1.28), and a moderate effect size was achieved on the SIAS-6/SPS-6, and PDSS-SR (*d* = .76 and d = .71, respectively). The within-group effect sizes appeared stable through to 3-month follow-up.

At pre-treatment, 71/89 (80%, 95% CI (70–87%) CL+CO participants scored above the cutoff for the GAD-7 (total score ≥8). At post-treatment 46/71 (65%, 95% CI (53–75%) met criteria for remission (GAD-7 total score <7) and 36/71 (51%, 95% CI (39–62%) met criteria for recovery (GAD-7 total score <7 and reduction of at least 50% in total score). At 3-month follow up, 45/71 (63%, 95% CI (52–74%) met criteria for remission and 37/71 (52%, 95% CI (70–87%) met criteria for recovery. Additionally, at 3-month follow-up, 46/89 (52%, 95% CI (41–62%) of the CL+CO group no longer met diagnostic criteria for a principal diagnosis of GAD, SP or Pan/Ag (Table 3). Chi square tests demonstrated a significant reduction from pre-treatment to 3-month follow-up in the number of participants meeting criteria for GAD (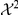
(1, N = 178) = 13.92, *p*<.05), SP (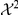
(1, N = 178) = 5.75, *p*<.05), and a non-significant reduction regarding Pan/Ag (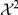
(2, N = 178) = 3.16, *p* = .08).

Thirty-seven of 43 (86%) CO and 40/46 (87%) CL group participants completed post-treatment satisfaction questionnaires. Results for the two groups were pooled as there were no significant differences in satisfaction ratings (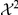
(2, N = 78) = 4.81, *p* = .09). Sixty-five of seventy-seven (84%) CL+CO group participants responded to the satisfaction questionnaire and reported that they were either *very* or *mostly satisfied* with the Program. An additional 12/77 (16%) participants reported they were *neutral/somewhat dissatisfied* with the Program, but no participants reported they were *very dissatisfied* with the Program. Additionally, 75/77 (97%) participants said they would feel confident in recommending the Program to a friend.

### Does the program result in change in each specific disorder?

Pre-treatment, post-treatment, and 3-month follow-up data for the pooled CL+CO group by principal disorder is presented in Table 6. Pre to post-treatment paired sample t-tests revealed significant improvements in PSWQ, SIAS-6/SPS-6 and PDSS-SR scores, regardless of principal diagnosis (*t _range 19-38_* = 3.65 – 9.13, *p*<.000). Importantly, these analyses were significant when adjusting for the multiple comparisons required to examine this effect across the nine questionnaire measures (*p* significance level = .005). Paired sample t-tests revealed no change on the PSWQ, PDSS-SR, or SIAS-6/SPS-6 from post-treatment to 3-month follow up for any of the three principal diagnoses (*t_ range 19-38_* = .11 – 1.53, *p* = 0.14 – .91).

**Table 6:** Means, standard deviations and effect sizes (Cohen's d) for pooled CL+CO group on disorder specific outcome measures at pre-treatment, post-treatment and 3-month follow-up timepoints.

Measure and Principal diagnosis	Timepoint				Within group effect size	
	n	Pre-treatment Mean	Post-treatment Mean	Follow-up Mean	Pre- to post-treatment	Pre-treatment to follow-up
**PSWQ**						
Total	89	63.63 (11.01)	52.07 (10.70)	52.06 (13.37)	1.07 (−1.22 –3.29)	0.95 (−1.34 – 3.73)
GAD	39	67.38 (10.43)	54.77 (10.23)	54.44 (13.08)	1.24 (−2.04 – 4.45)	1.11 (−2.17 – 5.21)
SP	30	58.73 (11.22)	47.57 (10.70)	48.20 (11.64)	1.04 (−2.98 – 4.86)	0.94 (−3.08 – 5.10)
Pan/Ag	20	63.65 (9.21)	53.55 (9.82)	53.20 (15.58)	1.09 (−2.95 – 5.39)	0.84 (−3.20 –7.67)
**SIAS-6/SPS-6**						
Total	89	20.31 (11.45)	12.56 (9.03)	13.26 (10.53)	0.76 (−1.62 – 2.63)	0.64 (−1.73 – 2.83)
GAD	39	17.85 (11.32)	10.79 (9.28)	10.92 (9.92)
SP	30	25.10 (10.29)	15.97 (8.52)	15.73 (9.48)	0.98 (−2.70 – 4.03)	0.96 (−2.72 – 4.36)
Pan/Ag	20	17.95 (11.61)	10.90 (8.17)	14.10 (12.59)	0.72 (−4.37 – 4.30)	0.33 (−4.76 – 5.84)
**PDSS-SR**						
Total	89	10.20 (6.89)	5.71 (5.80)	5.97 (7.31)	0.71 (−0.72 – 1.91)	0.60 (−0.83 – 2.12)
GAD	39	8.97 (6.79)	5.38 (5.27)	4.77 (6.80)	0.60 (−1.53 – 2.25)	0.63 (−1.50 – 2.76)
SP	30	7.90 (5.27)	3.87 (4.57)	4.00 (4.79)	0.83 (−1.05 – 2.47)	0.79 (−1.10 – 2.50)
Pan/Ag	20	16.05 (6.13)	9.10 (7.13)	11.25 (9.03)	1.07 (−1.61 – 4.20)	0.64 (−2.05 – 4.60)

*Note.* The standard deviations of the means and the confidence intervals of effect sizes are shown in parentheses. Intention-to-treat model was employed with pre-treatment scores being carried forward if post-treatment or follow-up data was not available Abbreviations: PSWQ: Penn State Worry Questionnaire; SIAS-6/SPS-6: Social Interaction Scale and Social Phobia Scale Short Form; PDSS-SR: Panic Disorder Severity Scale – Self Rating.

Participants with a principal diagnosis of GAD, SP or Pan/Ag achieved large within-group effect sizes on their corresponding disorder-specific measure (Table 6). Additionally, participants achieved small to large effect sizes on disorder-specific measures that did not correspond to their principal diagnosis. These gains were generally stable at 3-month follow-up.

### Can good clinical outcomes be obtained when support is provided by a Coach?

Pre-treatment, post-treatment and 3-month follow up data for the CO and CL groups is presented in Table 5. Univariate ANCOVAs controlling for pre-treatment scores revealed the CO group had significantly lower GAD-7 scores, and a trend towards significantly lower DASS-21 scores, than the CL group at post-treatment (*F_1,88_* = 5.37, *p* = .02, *F_1,88_* = 3.85, *p* = .05, respectively). There was no significant difference between CO and CL groups on PSWQ, SIAS-6/SPS-6, PDSS-SR, PHQ-9 and SDS at post-treatment (*F_1, 88_* = 1.0 – 3.72, *p* = .06 – 32).

Univariate ANCOVAs controlling for pre-treatment scores revealed the CO group had significantly lower GAD-7, PDSS-SR, PHQ-9, and SDS scores than the CL group at 3-month follow up (*F_1, 88_* = 5.11 – 7.71, *p* = .007 – .03), but no difference on the DASS-21, PSWQ, SIAS-6/SPS-6, (*F_1, 88_* = 1.85 – 2.94, *p* = .09 – .18). Paired samples t-tests revealed no significant change from post-treatment to 3-month follow up on any measure for either CO (*p* range = .69–1.0) and CL (*p* range = .25–.48) groups.

Between- and within-group effect sizes on primary, disorder-specific measures, and measures of depression and disability are included in Table 5. Small to medium (*d* = .20 –.62) between-group effect sizes were achieved by the CO group relative to the CL group on all measures at post-treatment and 3 month-follow-up. Large within-group effect sizes were achieved by the CO group on all measures at post-treatment (*d* = .80 – 1.29). At post-treatment, the CL group achieved large within group effect sizes on the DASS-21, PSWQ, and SDS (*d* = .81 – .90), and moderate effect sizes on the GAD-7, SIAS-6/SPS-6, PDSS-SR, and PHQ-9 (*d* = .67 – .71). These gains appeared generally stable at 3-month follow-up.

### Contact Events

Table 1 displays the frequency of contact events and duration of total contact time per participant. No significant differences were observed between the CO and CL groups in the number of phone calls, manually written contacts, automated written contacts or the total contact time provided by the Coach and Clinician throughout the program (*t_87_* range = −1.79 – .87, *p* range = .08 – .98).

### Control group results

As a partial replication of the CO condition, Control group participants received weekly support from the Coach during their treatment phase, consistent with that provided to the original CO group. One Control group participant withdrew before beginning the active treatment phase of the program reporting their symptoms had sufficiently resolved, and another could not be contacted, resulting in 40 Control group participants commencing the active treatment phase of the Anxiety program and eligible for analysis. Of these, 33 (82.5%) participants completed the eight lessons within the ten weeks of the program, and an additional two (5%) participants completed the remaining lesson within seven days of the program ending. The average number of Lessons completed was 7.56 (SD = 1.19). Post-treatment data was collected from 38/40 (95%) Control group participants.

Post-treatment Control group results and within-group effect sizes on primary, disorder-specific outcome measures, and measures of depression and disability are presented in Table 4. Paired samples *t*-tests revealed that the Control group achieved significant reductions from pre- to post-treatment on all measures (t_39_ range = 5.06 – 9.46, all *p<*.000). Importantly, these analyses were significant when adjusting for the multiple comparisons required to examine this effect across the seven questionnaire measures (*p* significance level = .007). The Control group achieved within-group effect sizes consistent with the original CO group, on all measures at post-treatment. Thirty-one of the 37 (84%) control group participants who completed the post-treatment satisfaction questionnaires reported being either *very satisfied* or *mostly satisfied* with the program, while six (16%) participants reported being *neutral/somewhat dissatisfied* with the Program, and no participants reported feeling *very dissatisfied* with the program. Additionally, 36/37 (97%) participants said they would feel confident in recommending the program to a friend.

## Discussion

This trial examined the efficacy of an extended version of the Anxiety program, a transdiagnostic iCBT program for anxiety disorders, when guided by either a Coach or Clinician. At intake all participants met DSM-IV diagnosis for generalized anxiety disorder, social phobia, or panic disorder (with or without agoraphobia) and 70% met criteria for at least one additional disorder.

### Is a transdiagnostic iCBT program for anxiety disorders efficacious?

Outcomes for the pooled treatment groups (CL+CO) were superior to the Control group on all measures and this was associated with large between-group effect sizes, with the exception of the SDS where a moderate effect size was obtained. Follow-up data indicated treatment effects were maintained. At follow-up more than half the CL+CO group did not meet criteria for their principal diagnosis. Adherence and satisfaction with treatment was high, suggesting that transdiagnostic approaches are acceptable to consumers. Importantly, these results were obtained with less than 70 min of total Clinician or Coach time per participant, and appear consistent with outcomes achieved in the low intensity treatments offered in recent field trials of the UK based Improved Access to Psychological Therapy program [4].

### Does the program result in change in each specific disorder?

Significant reductions were found on the corresponding disorder-specific outcome measure for participants with each of the three principal diagnoses. Within-group effect sizes for each of the target disorders on their corresponding disorder-specific measure were large and gains were maintained at follow-up. Participants also achieved significant reductions on disorder-specific measures different to their principal diagnosis.

### Can good clinical outcomes be obtained when support is provided by a Coach?

With one exception, no significant differences were found between the CL and CO groups at post-treatment, the exception being a lower GAD-7 score in the CO group. At follow-up the CO group had significantly lower symptom severity scores than those in the CL group on the GAD-7, PDSS-SR, and SDS. These results were unanticipated, and require replication. As a partial replication, the Control group received the CO treatment and achieved post-treatment outcomes comparable to those obtained by the original CO group. This result is consistent with studies indicating that non-clinical support roles for guided and highly structured iCBT programs for common mental disorders are associated with good clinical outcomes [9], [11], [49].

### Limitations

Limitations of the present study are relevant to other studies in the field of transdiagnostic treatment. We had sufficient power to detect medium to large differences between groups, but not to detect small differences between groups or to compare between groups based on principal diagnosis. Consequently, small differences between groups could exist that were not detected in the present study, and which future studies employing larger sample sizes may reveal. It is important to note that the sample sizes required to address these issues are considerable, and was only able to be approximated by pooling treatment data. Pragmatic approaches such as those used here may provide a practical and preliminary alternative for answering such important questions; however, more expansive research is required. Future research employing larger samples may benefit from considering mixed models approaches that will further inform the debate surrounding treatment response.

A second limitation concerns the choice of general and disorder specific outcome measures; an issue identified in the broader field of transdiagnostic research [16]. In the present study we selected brief measures to reduce burden on participants. There is a need for broader discussion regarding the questionnaire batteries most appropriate for the evaluation of transdiagnostic treatment and to facilitate comparison of results.

A third limitation concerns blinding. Due to resource constraints researchers were not blinded for 3-month follow-up diagnostic interviews, which may have resulted in under-reporting of diagnostic symptoms. The enduring gains made by treatment groups across a broad range of outcome measures mitigates some of this concern, however, future research will clearly benefit from blinding in diagnostic interviews.

A fourth limitation concerns the generalizability of the current findings. Independent replication is required to further understand the relative efficacy of Clinician and Coach roles for guided iCBT. Future studies would also benefit from comparing treatment with an active control group rather than a delayed-treatment waiting list. For example, the treatment gains in the present study may be solely due to telephone contact and, although unlikely, this cannot be ruled out using a wait-list control group. Future research employing telephone contact as part of an active control may go some way to informing non-specific treatment effects.

An additional limitation is the duration of follow-up analyses. Some authors argue that transdiagnostic treatments target underlying vulnerabilities and thus may lead to more durable treatment effects [50]. Follow-up data in the present study is consistent with existing research [51], yet future research would benefit from considering longer follow-up periods.

### General discussion

The findings of the current study are consistent with both the broader transdiagnostic and iCBT literature. Improvements on the general measures of anxiety are consistent with meta-analyses of transdiagnostic face-to-face programs for the anxiety disorders [52]. Analyses by principal disorder indicated improvement on the relevant diagnosis-specific outcome measures and also on diagnosis-specific measures different to the principal diagnosis. This supports the argument that transdiagnostic treatments may help consumers generalise beyond their principal complaint [16].

The magnitude of treatment gains in the present study are also comparable with those reported in meta-analyses of Internet and computer-aided psychotherapy for symptoms of anxiety [7]. Analyses by principal disorder yielded results consistent with those reported in recent studies of disorder-specific iCBT programs for GAD [53], SP [54] and Pan/Ag [46]. The outcomes achieved by the CO group, which were partially replicated with the Control group, are consistent with research indicating that coaching and clinical support roles can result in similar outcomes for guided iCBT [9], [45].

The Control group in the present research provided a partial replication of the CO condition, but independent replication of the study is required to examine the reliability of the findings. Future research examining the role of comorbidity and consumer attitudes, are two areas of research that will inform discussion regarding transdiagnostic treatment and are the topic of studies currently underway. Future research exploring the relative efficacy of transdiagnostic and individually-tailored interventions would be of value, as both approaches have provided encouraging findings regarding disorder specific change and have potential for the treatment of comorbidity [25], [55]. Additionally, future studies using a larger sample size would allow comparison of transdiagnostic iCBT with disorder-specific iCBT, and would begin to inform the debate around the relative utility of these approaches. An unresolved tension in the field of transdiagnostic treatment concerns the suitability of disorders such as OCD and PTSD to this approach [17], and inclusion of a broader range of disorders is required for future research to begin answering these questions. Moreover, research examining the relative benefits of clinician and coaching guidance in non-research environments is required to inform discussion about the dissemination of low-intensity interventions [5], [56].

### Conclusions

This is an extension of a transdiagnostic iCBT program for three anxiety disorders. This randomized controlled trial revealed overall outcomes that were superior for the treatment groups relative to a waitlist control condition and which were stable over a 3 month follow up period and satisfactory to participants. Outcomes by principal diagnosis appeared consistent with those obtained in disorder-specific iCBT programs, and allowed participants to generalise gains beyond symptoms of their principal complaint. Coach assisted iCBT was as effective as Clinician assisted iCBT. Further studies need to explore questions about the role of comorbidity, consumer attitudes, to investigate clinical and coaching support roles and the relative efficacy of transdiagnostic and disorder-specific iCBT.

## Supporting Information

Checklist S1 CONSORT checklist.
(DOC)

Protocol S1 Trial protocol.
(DOCX)
