# Supplementary material for: A RCT of a Transdiagnostic Internet-Delivered Treatment for Three Anxiety Disorders: Examination of Support Roles and Disorder-Specific Outcomes
Source: PLoS One. 2011 Nov 23;6(11):e28079. doi: 10.1371/journal.pone.0028079 (PMC3223218; doi:10.1371/journal.pone.0028079)
Supplement: Protocol S1 — Trial protocol. (DOCX) [file pone.0028079.s002.docx]

**Protocol Title**

**Internet Based Transdiagnostic Treatment for Anxiety - A Randomised Controlled Trial of the Anxiety Program**

HREC Ref:

SVH Ref:

Version Number: 3

Date of Protocol: 09/03/2010

SYNOPSIS

Protocol title: Internet Based Transdiagnostic Treatment for Anxiety - A Randomised Controlled Trial of the Anxiety Program

Protocol version: 3

LIST OF INVESTIGATORS

Chief Investigator: N Titov

Address: Clinical Research Unit for Anxiety and Depression, UNSW at St Vincent’s Hospital

Telephone no.: +612 8382 1732

Fax no.: +612 8382 1721

Principal Investigator: J G Andrews

Address: Clinical Research Unit for Anxiety and Depression, St Vincent’s Hospital

Telephone no.: 8382 1726

Fax no.: 83821721

Associate Investigator: E Robinson

Address: Clinical Research Unit for Anxiety and Depression, St Vincent’s Hospital

Telephone no: 8382 1729

Fax no.: 83821721

Associate Investigator: J Spence

Address: Clinical Research Unit for Anxiety and Depression, St Vincent’s Hospital

Telephone no: 8382 1729

Fax no.: 83821721

Associate Investigator: L Johnston

Address: Clinical Research Unit for Anxiety and Depression, UNSW St Vincent’s Hospital

Telephone no: 8382 1725

Fax no.: 83821721

**Summary**

Protocol title: Internet Based Transdiagnostic Treatment for Anxiety - A Randomised Controlled Trial of the Anxiety Program

Protocol version: 2

Objectives Primary objective: To explore the outcomes of the Anxiety program for participants with either Generalised Anxiety Disorder (GAD), social phobia, or panic disorder (with or without agoraphobia), who receive clinician assisted computerized cognitive behavioural therapy (CaCCBT), against participants who receive computerized CBT with reminder calls from administrative staff (CCBT), against participants who remain on the wait list, using a randomized controlled trial design.

Secondary objectives: To further determine the feasibility of these treatments in terms of acceptability to patients and practicality for clinicians.

Study design: A CONSORT-R compliant, registered RCT of the intervention (CaCCBT) versus CCBT versus waitlist control group.

Planned sample size: 150

Selection criteria: Meets criteria for GAD, social phobia, or panic disorder (with or without agoraphobia) as a primary diagnosis.

Study procedure Recruit and treat subjects with GAD, social phobia or panic disorder (with or without agoraphobia)

Statistical considerations: Sample size calculation: Assuming ES > 0.5

Analysis plan: ANCOVA of post-treatment scores on measures of depression, anxiety, disability, and treatment satisfaction, while controlling for pre-treatment score. T-tests and *X*^2^ on pre-treatment scores to determine equivalence between groups.

Duration of the Study: 18 months

TABLE OF CONTENTs PAGE

1. BACKGROUND 6

1.1 Disease Background 6

1.2 Rationale for Performing the Study 7

2. STUDY OBJECTIVES 7

2.1 Primary Objective 7

2.2 Secondary objectives 8

3. STUDY DESIGN 7

3.1 Design 7

3.2 Study Groups 8

3.3 Number of Participants 8

3.4 Number of Centres 8

3.5 Duration 8

4. PARTICIPANT SELECTION 8

4.1 Inclusion Criteria 8

4.2 Exclusion Criteria 8

5. STUDY OUTLINE 9

5.1 Study Flow Chart 9

5.2 Investigation Plan 10

5.3 Study Procedure Risks 12

5.4 Recruitment and Screening 12

5.5 Informed Consent Process 12

5.6 Enrolment Procedure 12

5.7 Randomisation Procedure 12

6. SAFETY 12

6.1 Adverse Event Reporting 12

6.2 Serious Adverse Event Reporting 13

6.3 Data Safety and Monitoring Board - membership and responsibilities 13

6.4 References to national and international guidelines on research in humans 13

6.5 Early Study Termination 13

7. BLINIDING AND UNBLINDING 13

8. STATISTICAL CONSIDERATIONS 13

8.1 Sample Size Calculation 13

8.2 Analysis Plan 14

9. STORAGE AND ARCHIVING OF STUDY DOCUMENTS 14

10. REFERENCES 14

11. APPENDICES 15

11.1 Comment on advertising of studies 15

11.2 Diagnostic Interview to be used 15

11.3 List of questionnaires to be used 16

11.4 Random Number Generator 24

# BACKGROUND

## Disease Background

There is increasing evidence that anxiety and depression share a common etiology. Goldberg et al, (in press), points to a number of shared characteristics including heritability, familiality, course, response to treatment and vulnerabilities between the disorders as evidence that what the disorders have in common outweighs what they do not.

This approach has sparked interest in the field of treatment for these disorders. The approach of treating what is common between disorders, often referred to as transdiagnostic treatment, has received growing attention (e.g. Page & Hooke, 2003; Lumpkin et al, 2002; Norton, 2008). Recent meta analyses of transdiagnostic treatment for the anxiety disorders (Norton & Price, 2007), and for anxiety combined with depression (McEvoy et al, 2009), has supported the efficacy of this approach, suggesting equivalence between disorder-specific and transdiagnostic treatments for the common mental disorders.

Despite the existence of effective disorder specific and transdiagnostic treatments, nearly two thirds of people with a mental disorder do not seek help, and only 22% of people with an anxiety disorder utilized a mental health service (ABS, 2009). The reason for the shortfall is well known as being a mixture of societal, attitudinal and diagnostic variables (Andrews et al, 2001). Making treatment more freely available in areas where expert treatment is not available, or to people who are unable to take time off work to access treatment is one logical step to improving access to effective healthcare.

We recently completed randomised controlled trials of clinician assisted computerised cognitive behavioural therapy (CaCCBT) for the distance treatment of people with social phobia (Titov et al, 2008a), GAD (Titov et al, 2009a), and panic disorder (Wims et al, 2008). Adherence across all the studies was good (social phobia, 80%; GAD, 75%, panic disorder 88%). Patients in the intervention groups were severe and yet made significant improvement (social phobia, ES=1.2 ; GAD, ES=1.3; Panic, ES=1.0). This level of improvement is roughly equivalent to that seen in our face-to-face anxiety disorders clinic at St Vincent’s hospital. However, the CaCCBT groups only required roughly one quarter as much clinician time as usual face to face treatment. This finding has been replicated for the social phobia program (Titov et al, 2008b), and replication studies for the GAD and depression programs will be finalised by the end of the year. Moreover, preliminary results suggest that disorder specific programs are successful in reducing symptom severity of non-targeted disorders (Titov et al, 2009b).

We have recently completed an RCT pilot study of an Internet-based treatment transdiagnostic treatment for anxiety disorders (Titov, Johnston, Robinson & Andrews, in preparation). Attendance throughout the program was roughly equivalent to that of our previous Internet-based disorder specific programs (76%), and preliminary findings support the efficacy of this initiative.

We are interested in further investigating the efficacy of Internet-based treatments for a population of people suffering from at least one of the following disorders: GAD, social phobia or panic disorder (with or without panic disorder). Additionally, we are interested in assessing the level of support required to help people complete the program as independently as possible.

## Rationale for Performing the Study

Many people with anxiety do not access treatment, despite the availability of diagnosis specific and transdiagnostic face-to-face initiatives. Internet-based treatment may be one way of circumventing the barriers to effective mental health care. We have developed Internet based treatment programs for GAD, social phobia, and panic disorder, which have all suggested that this treatment modality is effective. We have recently completed a RCT pilot study of transdiagnostic treatment for anxiety disorders delivered via the web, and preliminary results suggest that the program is effective.

This proposed study will recruit 150 people with GAD, social phobia, or panic disorder and randomly allocate them to one of three groups: (1) Clinician guided internet-based treatment group, (2) Self-guided internet-treatment group, (3) Wait-list control group (who will receive treatment after the treatment groups have completed the program). We will measure changes in symptom level from the beginning of the 10 week program course of treatment and one week after the conclusion of treatment, and at 3 month follow up. Measurement in the control group will be matched to the pre and post treatment assessment. We will also measure adherence to the lessons, homework, satisfaction with the clinician's input and satisfaction with the mode of treatment generally.

This proposed project employs the same research design as that previously approved by St Vincent's Hospital Human Research Ethics Committee (09/021). However, one additional questionnaire has been added for assessing general anxiety (Depression, Anxiety, Stress Scale, DASS-21, Lovibond & Lovibond, 1995), two questionnaires for diagnosis specific measures of anxiety (Social Phobia Screening Questionnaire, SPSQ, Furmark et al, 1999; Panic Disorder Severity Scale-Self Rating, PDSS-SR, Houck et al, 2002) and two questionnaires for associated variables (NEO-FFI, Costa & McCrae, 1992; Locus of Control Behaviour, Craig et al., 1984).

# STUDY OBJECTIVES

## Primary Objectives

The primary objectives of the study is comprised of three hypotheses: 1) That participants in treatment groups 1 and 2 would show significant improvement on a general measure of anxiety (DASS-21) and on disorder specific measures (GAD-7, PSWQ, SPSQ, PDSS-SR) relative to Controls; 2) that participants in treatment groups 1 and 2 with primary diagnoses of GAD, panic, or social phobia would show significant improvement on relevant disorder specific measures (GAD-7, PSWQ, SPSQ, PDSS-SR) relative to controls with the same primary diagnoses; and 3) that treatment groups 1 and 2 will be equivalent regarding generic and diagnosis specific measures of anxiety.

## Secondary objectives

The secondary objective is comprised of one hypothesis: that participants in treatment groups 1 and 2 will rate the program as acceptable.

# STUDY DURATION

## Design

CONSORT compliant Randomised Controlled Trial

## Study groups

Three study groups. 1) Clinician assisted computerized CBT (CaCCBT), 2) computerized CBT with reminder calls from administrative staff (CCBT), 3) waitlist control.

## Number of participants

150 in total: CaCCBT: n=50; CCBT: n = 50; Waitlist: n= 50

## Number of centres

1 – Clinical Research Unit for Anxiety and Depression, St Vincent’s Hospital, Sydney

## Duration

1^st^ February, 2010 to 1^st^ August, 2011, inclusive of recruitment, follow up and write up of results. The primary end point will be the end of active treatment for treatment groups 1 and 2 (i.e. 10 weeks after beginning the program) and the secondary time point will be the longer term follow up of these participants (i.e 3 months after completing active treatment).

# Participant section

## Inclusion criteria

Randomised Controlled Trial

The criteria for diagnosis of GAD, social phobia, or panic disorder (with or without agoraphobia) will be based on the Mini International Neuropsychiatric Interview Version 5.0.0 (Appendix A), which is based on DSM-IV diagnostic criteria.

Aged over 18, self identified as suffering from GAD, social phobia or panic disorder (with or without agoraphobia) and have questionnaire scores and results of telephone diagnostic interview consistent with this. Prepared to provide name, phone number and address, and to provide the name and address of a general practitioner and to provide written informed consent. Have access to a phone and computer with a printer.

## Exclusion criteria

Psychosis, bi-polar disorder, substance abuse or dependence disorders, severe depression and or/ current suicidality, or any biological disorder requiring immediate attention.

# Study OUTLINE

## Study Flow Chart

Enrolment (Feb 2010-March 2010)

Randomisation

Pre-treatment “Pre-treatment” assessment

assessment (April) time point (April)

CaCCBT and CCBT Waitlist control

Treatment Phase (10 weeks including “mid treatment

(10 weeks including mid assessment” time point)

treatment assessment)

Post-treatment assessment Pre-treatment assessment (begin

(June) active treatment, June)

Treatment Phase(10 weeks)

3 month follow up (September) Post-treatment assessment (August)

Writeup (2011)

##

## Investigation plan

A CONSORT-R compliant, registered RCT of the intervention (CaCCBT) versus CCBT versus waitlist control group. This will demonstrate whether the benefit from the CaCCBT intervention is superior to natural remission and placebo response. Because of low adherence to CCBT we think that it is a proxy for a placebo treatment. As in any RCT accurate patient selection, good randomization, reliable and valid outcome measures, and low drop-out rates are critical. Analyses based on completers and on intention to treat will be performed.

There are already over 1000 people on the waitlist for Virtual Clinic. In addition, some media attention is expected to generate more publicity to attract people to the program. The study will be described on the intake website, [www.virtualclinic.org.au](http://www.virtualclinic.org.au). People will read the information about the studies and elect to continue or not. If they choose to apply for the study they will complete an automated screening questionnaire. Only people whose responses to the questions meet selection criteria and undergo a brief phone interview explaining the program will read the information sheet and return an electronic consent form and mail that to the investigators. This will trigger the phone interview in which the diagnosis will be confirmed using the Mini International Neuropsychiatric Interview Version 5.0.0 (Appendix A). Questions about the study will be answered and an offer of treatment made.

Patients will be randomly allocated to CaCCBT group (n=50), CCBT group (n=50), or the waitlist group (n=50). Randomization will be done via [www.random.org](http://www.random.org) (see appendix A).

Upon beginning treatment, all participants will complete a set of diagnostic questionnaires. The questionnaires being administered in this study pre and post are the same as the study previously approved by St Vincent's Hospital Human Research Ethics Committee (09/021). However, one additional questionnaire has been added for assessing general anxiety (Depression, Anxiety, Stress Scale, DASS-21, Lovibond & Lovibond, 1995), three questionnaires for diagnosis specific measures of anxiety (Social Interaction Anxiety Scale/Social Phobia Scale Composite Short Form, SPSR, Peters et al, submitted,; Agoraphobic Cognitions Questionnaire, Chambless et al, 1984; Panic Disorder Severity Scale-Self Rating, PDSS-SR, Houck et al, 2002) and one questionnaires for associated variables (NEO-FFI, Costa & McCrae, 1992).

For the treatment groups, Dr Emma Robinson and Mr Jay Spence will treat the participants. The treatment program is delivered via the website [www.virtualclinic.org.au](http://www.virtualclinic.org.au). The treatment program consists of 8 lessons conducted over a 10 week period, in which participants are encouraged to complete one lesson per week. The program is comprised of the following Lessons:

**Lesson One:**

- **Learn about the physical, cognitive and behavioural symptoms of anxiety.**
- **Learn about treatments for anxiety**

**Lesson Two:**

- **Learn about the relationship between the way you think and feel.**
- **Begin monitoring and challenging unhelpful thinking patterns.**

**Lesson Three:**

- **Learn about your body and anxiety**
- **Controlling physical symptoms and maintaining physical balance**

**Lesson Four:**

- **Learn about behaviours that make anxiety worse**
- **Design a plan for confronting problems, slowly and gradually.**

**Lesson Five:**

- **Learn more about challenging unhelpful thinking patterns, and how to change the way you think**
- **Learn about structured problem solving**

**Lesson Six:**

- **Learn more about behaviours that make anxiety worse, and how troubleshooting common barriers to improvement**
- **Learn about how to put the skills together**

**Lesson Seven:**

- **Learn additional skills for tackling anxiety**
- **Learn about assertiveness and effective communication.**
- **Learn about the role of self-criticism in anxiety.**

**Lesson Eight:**

- **Revise the skills you have learnt.**
- **Make a plan for staying well in the future.**

Participants in the CaCCBT group will receive regular email contact with their clinician, and will be encouraged to carry out homework activities.The patients in the CCBT groups will have same access to the lessons, and homework activities as the CaCCBT groups. Unlike the CaCCBT groups, the CCBT groups will have no clinician contact over the 10 week period, but will receive weekly telephone reminders from administrative staff. The waitlist group will not receive any treatment during this period.

After the treatment group has finished the program, all participants will complete a third set of diagnostic questionnaires. The waitlist group, and any CCBT participants wishing to complete the program with clinician contact, will be offered additional treatment after this.

Effectiveness of the CaCCBT program for anxiety will be measured by comparing waitlist and CCCBT and CaCCBT groups on their pre- and post- treatment scores. Long-term follow up data will compare the CaCCBT and CCBT groups at 3 month follow up.

## Study Procedure risks

In applying to join the program, patients with severe depression/suicidality will be screened out and encouraged to see their GP. Patients will need to meet inclusion/exclusion criteria before joining the study. After satisfying these criteria, patients will be measured at pre-treatment, mid-treatment, post-treatment time points, with CaCCBT and CCBT groups measured at 3 month follow up and the waitlist control group measured immediately after completing the active treatment phase. If any responses to questionnaires at any of these timepoints indicate deterioration in wellbeing, this will be raised with the Chief Investigator, and or clinician to determine the best way of dealing with these difficulties. Additionally, regular team meetings (at least 3 times a week) will be conducted to monitor any difficulties patients may be having and ways of best dealing with these difficulties. Adverse events will be monitored by the investigators and reported to the Ethics Committees.

## Recruitment and screening

There are already over 1000 people on the waitlist for Virtual Clinic. The study will be described on the intake website, [www.virtualclinic.org.au](http://www.virtualclinic.org.au). People will read the information about the studies and elect to continue or not. If they choose to apply for the study they will complete an automated screening questionnaire to screen out participants with a history of schizophrenia/bi polar disorder, active suicidality, current substance use/dependence, or who do not meet the technical requirements of the study.

## Informed consent process

Only participants who have read the information about the studies, elected to complete the screening questionnaire, and meet selection criteria will be invited to participate. These participants will undergo a brief diagnostic interview, followed by further explanation of the study details, and an opportunity to ask questions about the study. A consent form will then be emailed to the participant. Informed consent will be taken as successfully returning the consent form. Additionally, informed consent will also be monitored during the study by the Chief Investigator, and each time a patient logs on to do a lesson, this will be taken as a sign of continued consent to participate.

## Enrolment procedure

Participants will be randomly allocated to Treatment Group 1, treatment Group 2, or the waitlist control group prior to completing the brief telephone interview.

## Randomization procedure

The website [www.random.org](http://www.random.org) will be used to determine a list of randomly generated numbers that correspond to a group (Group 1, 2 or waitlist control). Participants will be randomly allocated to one of these groups before completing the brief telephone interview.

# Safety

## Adverse event reporting

There are no known risks associated with this treatment. Adverse events will include events that prohibit study participants from accessing the Program. Regular team meetings (at least 3 times a week) will be conducted to monitor any difficulties patients may be having and ways of best dealing with these difficulties. Adverse events will be reported to the Ethics Committee.

## Serious adverse event reporting

There are no known risks associated with this treatment. Serious adverse events will be those indicating deterioration in wellbeing as per questionnaire responses at the time points throughout the program. Regular team meetings (at least 3 times a week) will be conducted to monitor any difficulties patients may be having and ways of best dealing with these difficulties. Serious adverse events will be reported to the Ethics Committee.

## Data safety and monitoring board

Data safety will comply with the National Statement on Ethical Conduct in Human Research. Only the responsible clinician and Chief Investigator will see identified data.

To maintain participant privacy and confidentiality, participants will not be able to use an alias/username that contains identifiable information (e.g real name, address, city you live in). If they choose an alias/username that contains identifiable information, they will be requested to change it before beginning the Program.

## Early termination

If a patient wishes to withdraw from the study once it has started, he or she can do so at any time without having to give a reason. Upon early withdrawal, all participants will be provided with encouragement to access additional services via their GP. Additionally, all participants will be given the opportunity to provide feedback about their current symptoms. If participants withdrawing from the program do report a deterioration of their symptoms, they will be contacted by the Chief Investigator to discuss access to additional services. If participants do not choose to provide feedback about their symptoms, but the investigators do have concerns about the participant’s condition, we do have consent from all participants to contact their GP. This process will be supervised by the Chief Investigator.

# Blinding and unblinding

Study is not blinded.

# STATISTICAL CONSIDERATIONS

## Sample Size Calculation

We would expect pre-post improvement of ES 1.0 for the CaCCBT groups on Anxiety Disorder measures. We also expect the CaCCBT group to improve more than the CCBT group by an ES of 0.3 and waitlist group by an ES of 0.6. Sample size is powered to have an 80% chance of detecting differences at p<.05.

## Analysis Plan

Analyses based on completers and on intention to treat will be performed. Three mixed design ANCOVAs will be carried out utilising SPSS GLM with the baseline score entered as a covariate. The within subject factor examines the difference in pre-post scores for each of the three treatment conditions, whilst the between subjects factor compares improvement scores between treatment groups.

# Storage and archiving of study documents

For the purpose of the study, participants will be registered as patients of St Vincent’s Hospital, Sydney. The process of managing this has been confirmed by representatives of both CRUfAD and St Vincent’s Hospital (see attached letter).

For the duration of the project the investigators will have access to individually identifiable data, to appropriately monitor participant progress. To do otherwise could compromise participant care. Following the completion of the project, only the Chief Investigator will have access to any participant data. This data will be re-identifiable for the purpose of any program audits and to respond to any participants who wish to apply for additional VirtualClinic Programs.

Please note that CRUfAD is relocating in April 2010. Throughout this process, any data associated with this process will be securely transferred and stored in the new site. Staff associated with the project are aware of, and will adhere to, the National Statement on Ethical Conduct in Human Research.

# REFERENCES

Andrews G., Issakidis, C., Sanderson, K., Corry, J., & Lapsley, H. (2004). Utilising survey data to inform public policy: comparison of the cost-effectiveness of treatment of ten mental disorders. *British Journal of Psychiatry, 184*, 526-533.

Australian Bureau of Statistics. (2009). *Australian Social Trends* (ABS cat. No. 4102.0), 1- 18.

Craig, A. R., Franklin, J. A., & Andrews, G. (1984). A scale to measure locus of control of behaviour. *British Journal of Medical Psychology*, *57,* 173–180.

Costa Jr., P.T., & McCrae, R.R. (1992). *Revised NEO Personality Inventory and five-factor inventory professional manual*. Psychological Assessment Resources, Odessa, FL.

Furmark, T., Tillfors, M., Everz, P. O., et al (1999) Social phobia in the general population: Prevalence and sociodemographic profile. *Social Psychiatry and Psychiatric Epidemiology*, *34*, 416 –424

Goldberg, D. P., Krueger, R. F., Andrews, G., & Hobbs, M. J. (in press). Emotional disorders: Cluster 4 of the proposed meta-structure for DSM-V and ICD-11. *Psychological Medicine*.

Houck, P.R., Spiegel, D.A., Shear, K., & Rucci, P. (2002). Reliability of the self-report version of the panic disorder severity scale. *Deperssion and Anxiety*, *15* (4), 183- 185.

Lovibond, S.H. & Lovibond, P.F. (1995). *Manual for the Depression Anxiety Stress Scales*. (2nd. Ed.). Sydney: Psychology Foundation.

Lumpkin, P., Silverman, W., Weems, C., Markham, M., & Kurtines, W. (2002). Treating a heterogeneous set of anxiety disorders in youths with group cognitive behavioral therapy: A partially nonconcurrent multiple-baseline evaluation. *Behavior Therapy, 33*(1), 163-177.

McEvoy, P. M., Nathan, P., & Norton, P. J. (2009). Efficacy of transdiagnostic treatments: a review of published outcome studies and future research directions *Journal of Cognitive Psychotherapy, 23*(1), 20-33.

National Health and Medical Research Council (NHMRC) (2007). *National* *Statement on Ethical Conduct in Human Research*. Canberra, NHMRC.

Norton, P. (2008). An open trial of a transdiagnostic cognitive-behavioral group therapy for anxiety disorder. *Behavior Therapy, 39*(3), 242-250.

Norton, P., & Price, E. (2007). A meta-analytic review of adult cognitive-behavioral treatment outcome across the anxiety disorders. *The Journal of nervous and mental disease, 195*(6), 521.

Page, A. C., & Hooke, G. R. (2003). Outcomes for depressed and anxious inpatients discharged before or after group cognitive behavior therapy: a naturalistic comparison. *The Journal of Nervous and Mental Disease, 191*(10), 653-659.

Titov, N., Andrews, G., Schwencke, G., Drobny, J., & Einstein, D. (2008a). “Shyness 1”, a randomized controlled trial of an Internet-based treatment for social phobia. Australian and New Zealand Journal of Psychiatry.

Titov, N., Andrews, G., & Schwencke, G. (2008b). Shyness 2: Treating social phobia online: Replication and extension. *Australian and New Zealand Journal of Psychiatry, 42*(7), 595-605.

Titov, N., Andrews, G., Robinson, E., Schwencke, G., Johnston, L., Solley, K., & Choi, I. (2009a). Clinician-assisted Internet-based treatment is effective for generalized anxiety disorder: randomized controlled trial *Australian and New Zealand Journal of Psychiatry, 43*(10), 905 – 912.

Titov, N., Gibson, M., Andrews, G., & McEvoy, P. (2009b). Internet treatment for social phobia reduces comorbidity. *Australian and New Zealand Journal of Psychiatry, 43*(8), 754-759.

Wims, E., Titov, N., & Andrews, G. (2008). The Climate Panic program: an open trial of Internet-based treatment for panic disorder. E-Journal of Applied Psychology, *4*(2), 26-32.

# APPENDICES

11.1 The study will not be advertised as we have over 1000 patients on the waiting list for a variety of Virtual Clinic programs. When the program becomes available, participants who have registered their interest in the VirtualClinic programs will receive a short email informing them that the program is open for applications.

11.2 Diagnostic Interview measure will be:

Mini International Neuropsychiatric Interview Version 5.0.0 (Sheehan, & Lecrubier, 2006)

This is a validated and widely used diagnostic measure and is widely used in psychological research.

11.3 Questionnaires to be included in the project are:

GAD-7: Short measure of Generalised Anxiety Disorder (Spitzer, Kroenke, Williams, & Löwe, 2006)

DASS-21: Depression, Anxiety, Stress Scales - 21 item version (Lovibond & Lovibond, 1995)

PSWQ: Penn State Worry Questionnaire (Meyer et al, 1990)

SPSR: Social Interaction Anxiety Scale / Social Phobia Scale Short Form Composite(Peters, et al, submitted)

ACQ; Agoraphobic Cognitions Questionnaire (Chambless et al, 1984)

PDSS-SR: Panic Disorder Severity Scale - Self Report Version (Shear et al 1999)

NEO-FF-I, N scale: NEO- Five Factor Inventory, Neuroticism scale (Costa & McRae, 1992)

PHQ-9: Patient Health Questionnaire – Nine item, (Kroenke, Spitzer, & Williams, 2001)

K-10: Kessler – 10 Item (Kessler, Andrews, Colpe et al, 2002)

SDS: Sheehan Disability Scale (Sheehan, 1983)

CEQ: Credibility/Expectancy Questionnaire (Devilly & Borkovec, 2000)

Each of these measures are validated and widely used in psychological research:

GAD-7


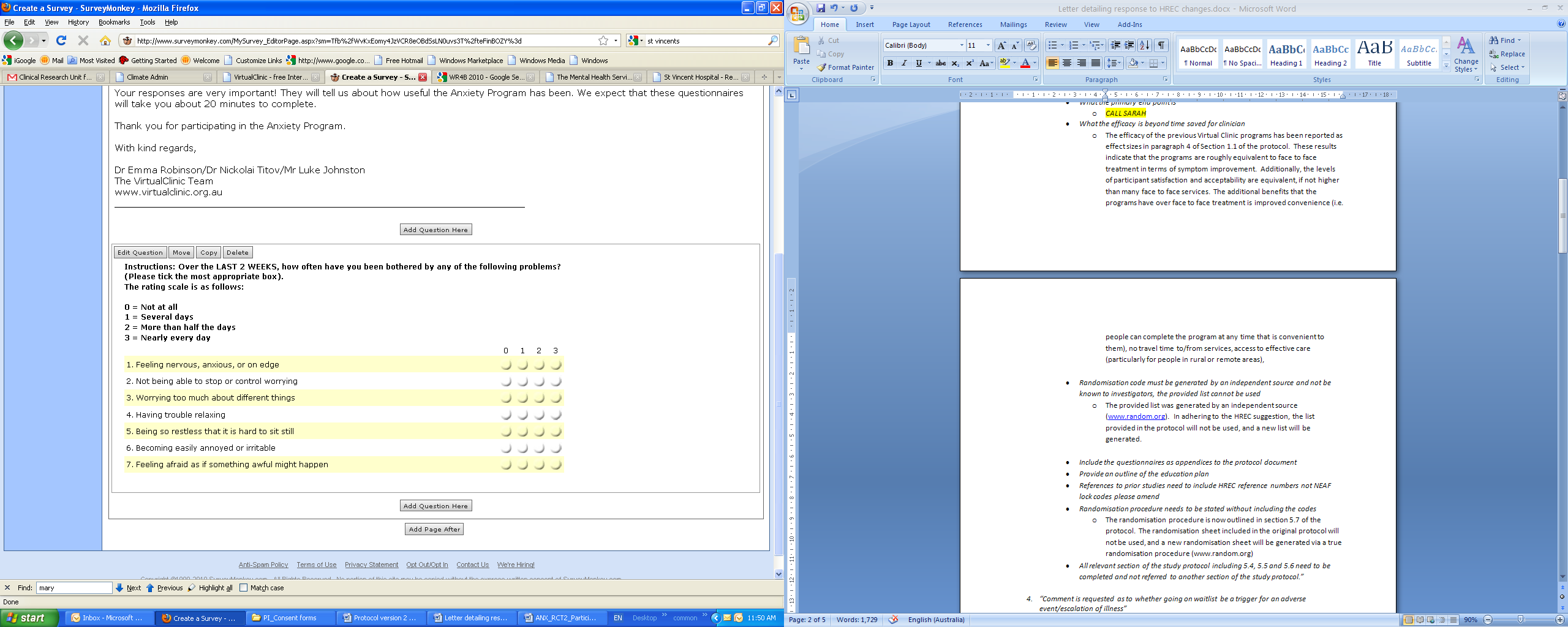


DASS-21


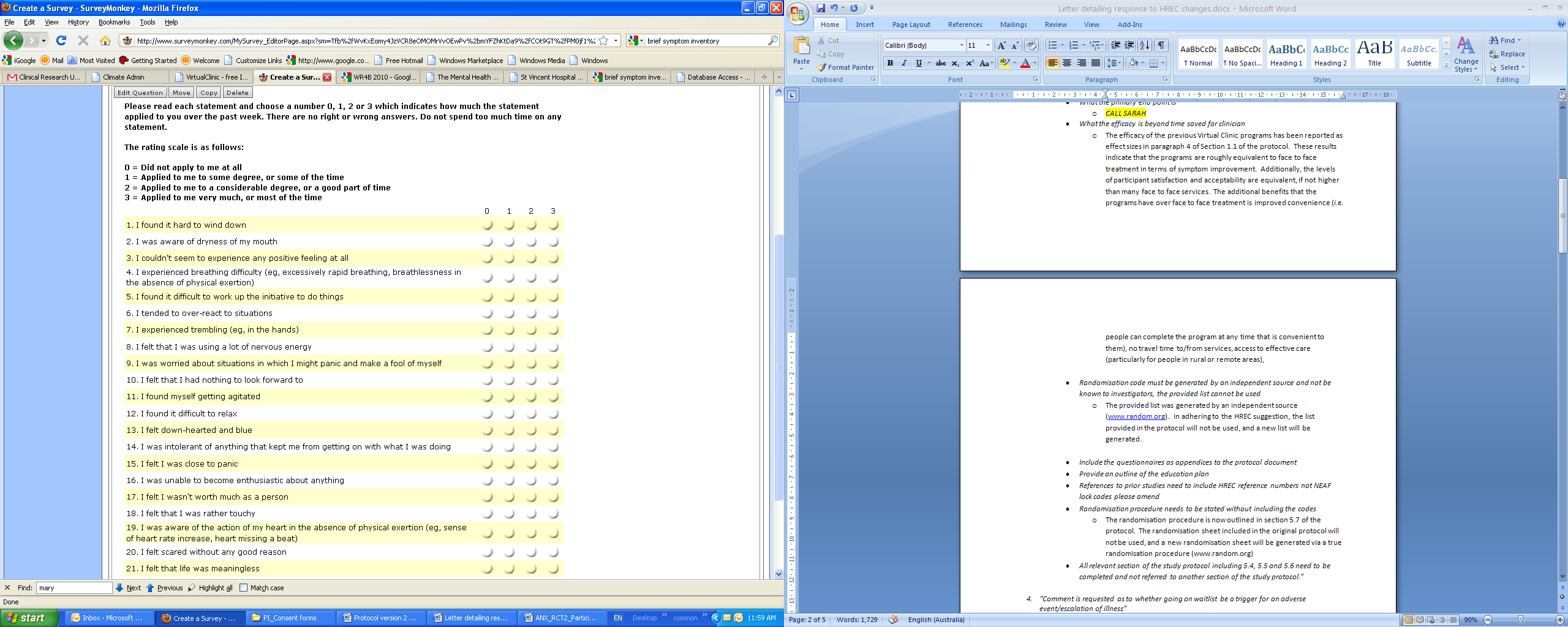


PHQ-9


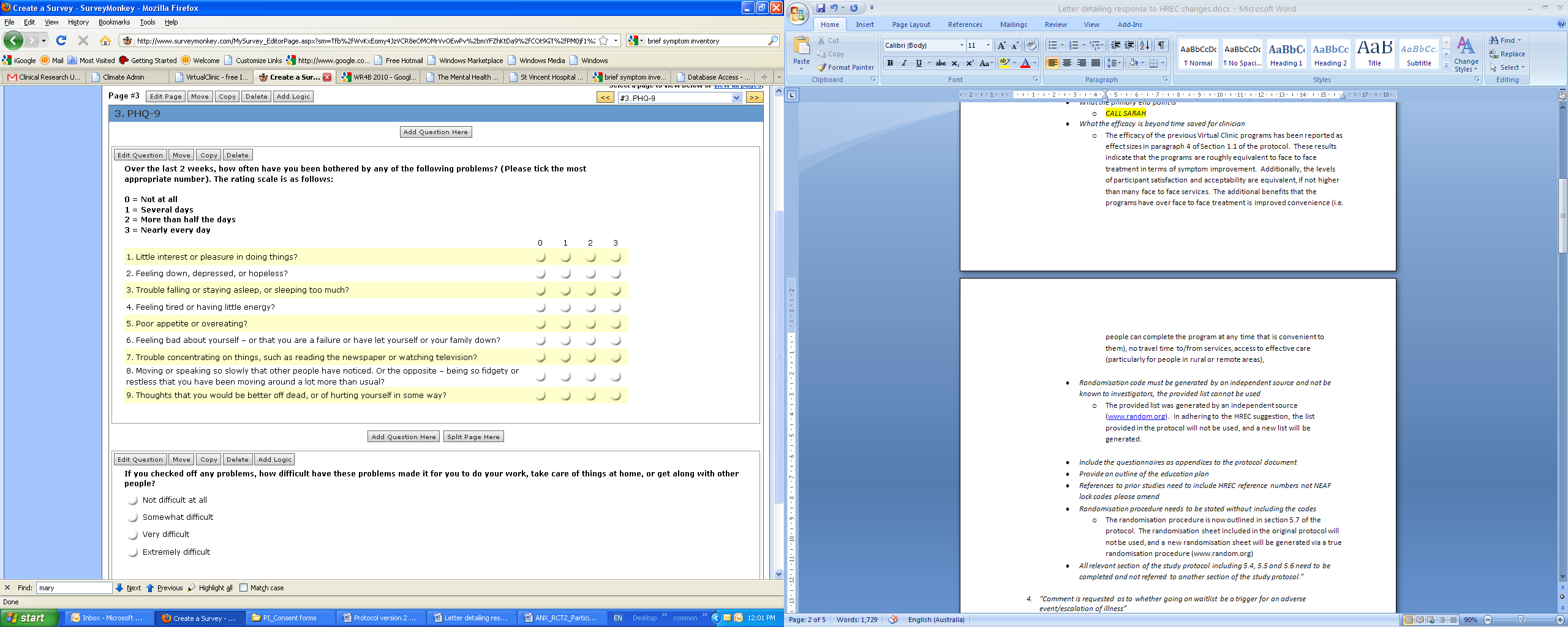


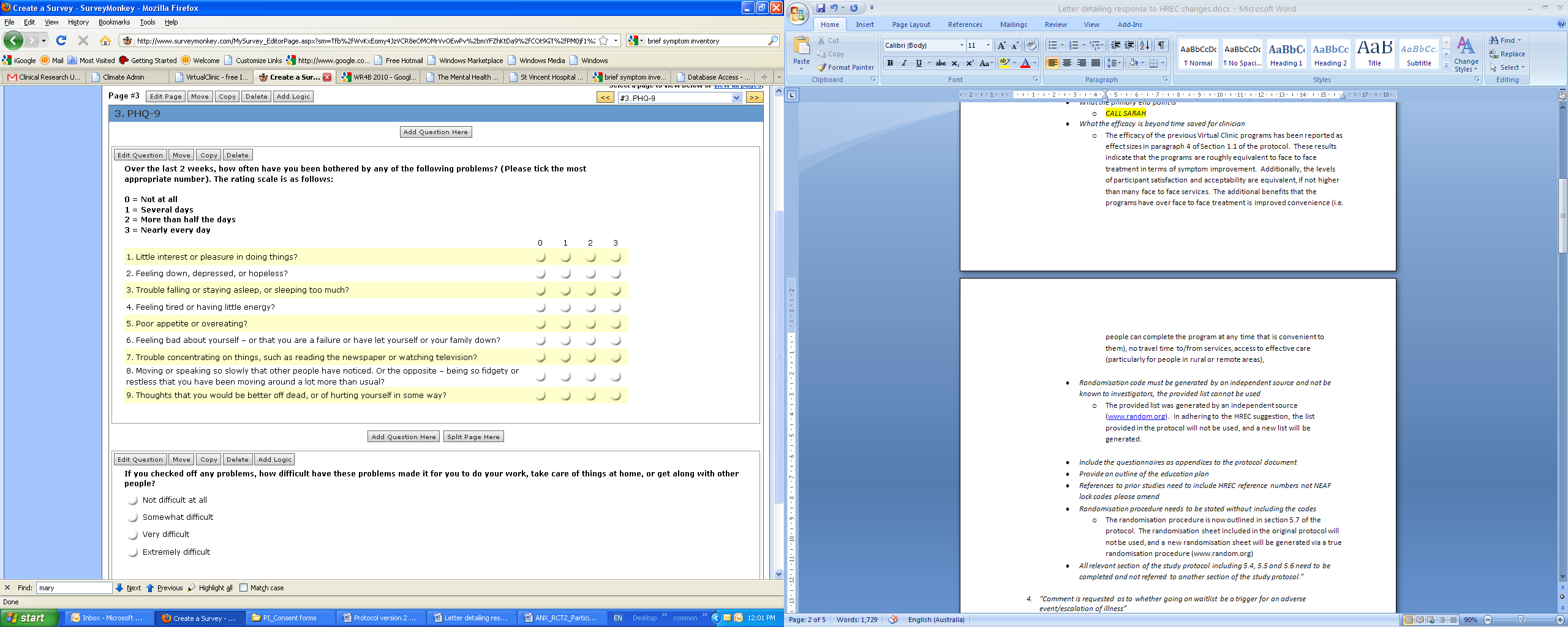


PSWQ


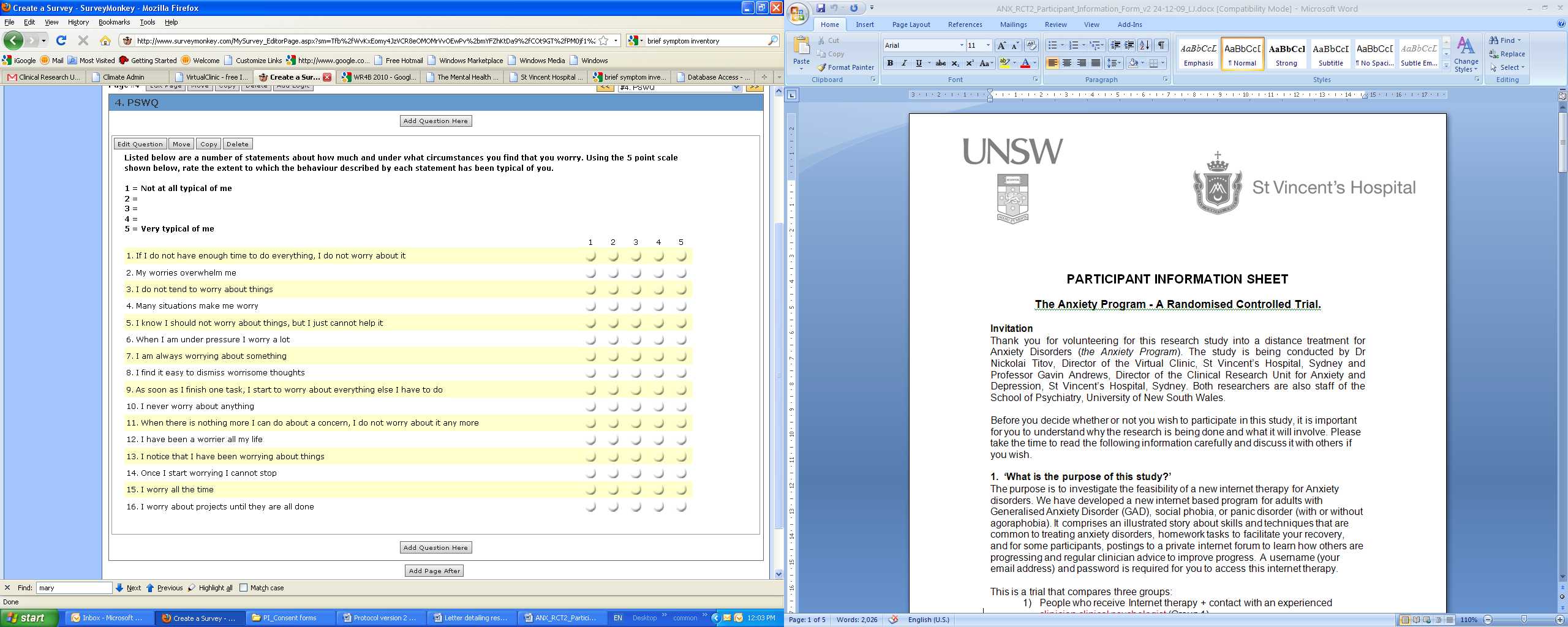


SPSR


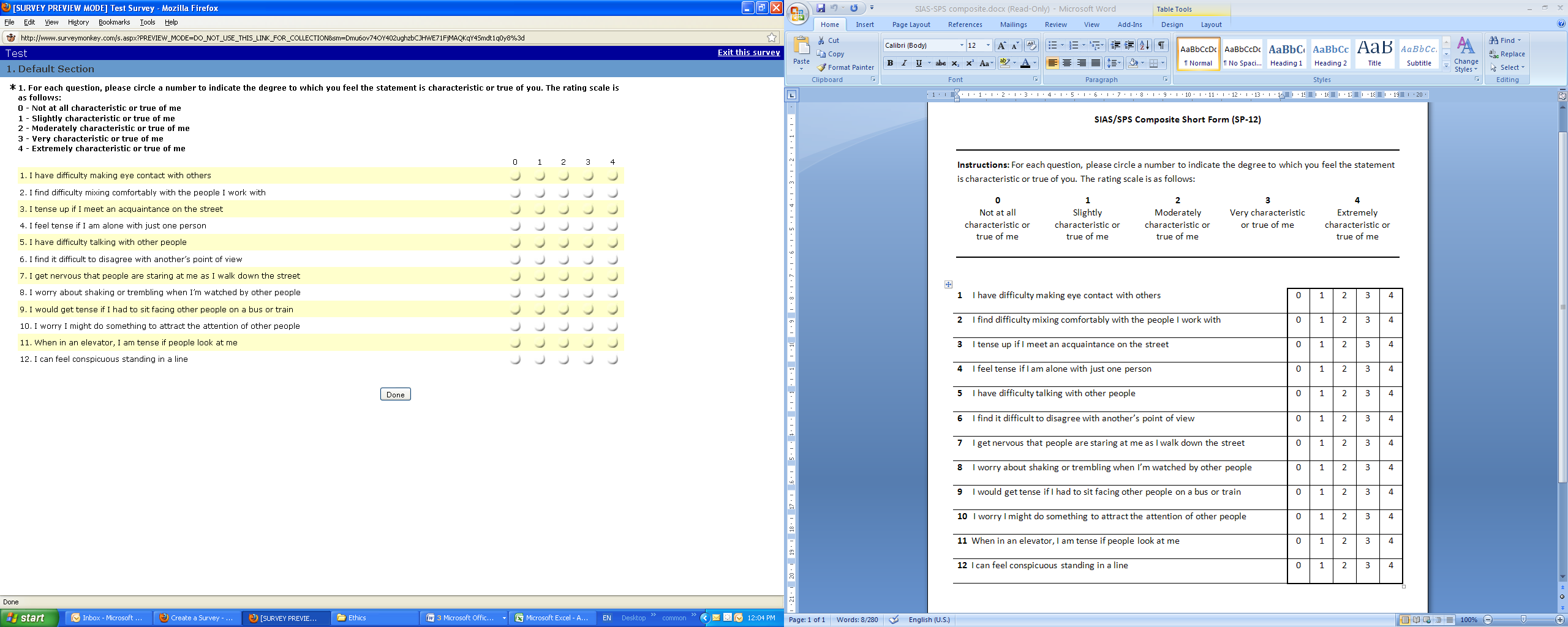


ACQ


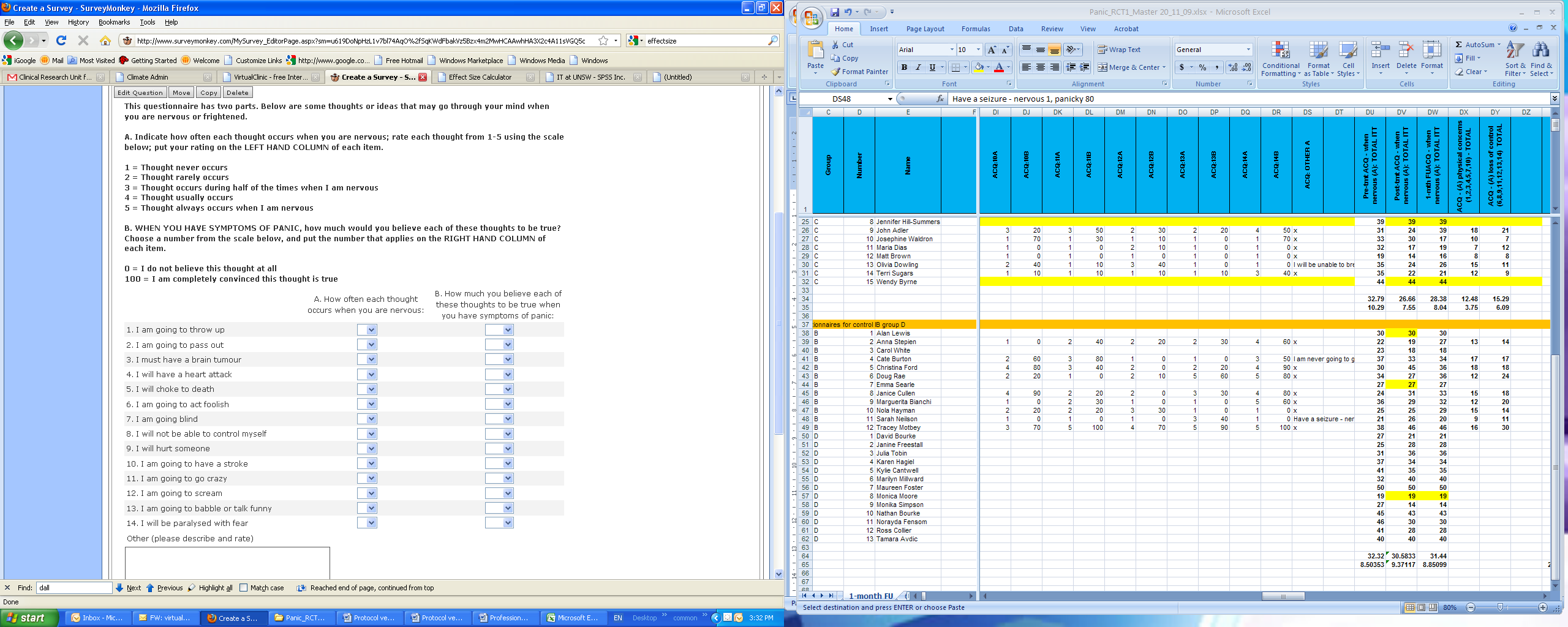


PDSS-SR


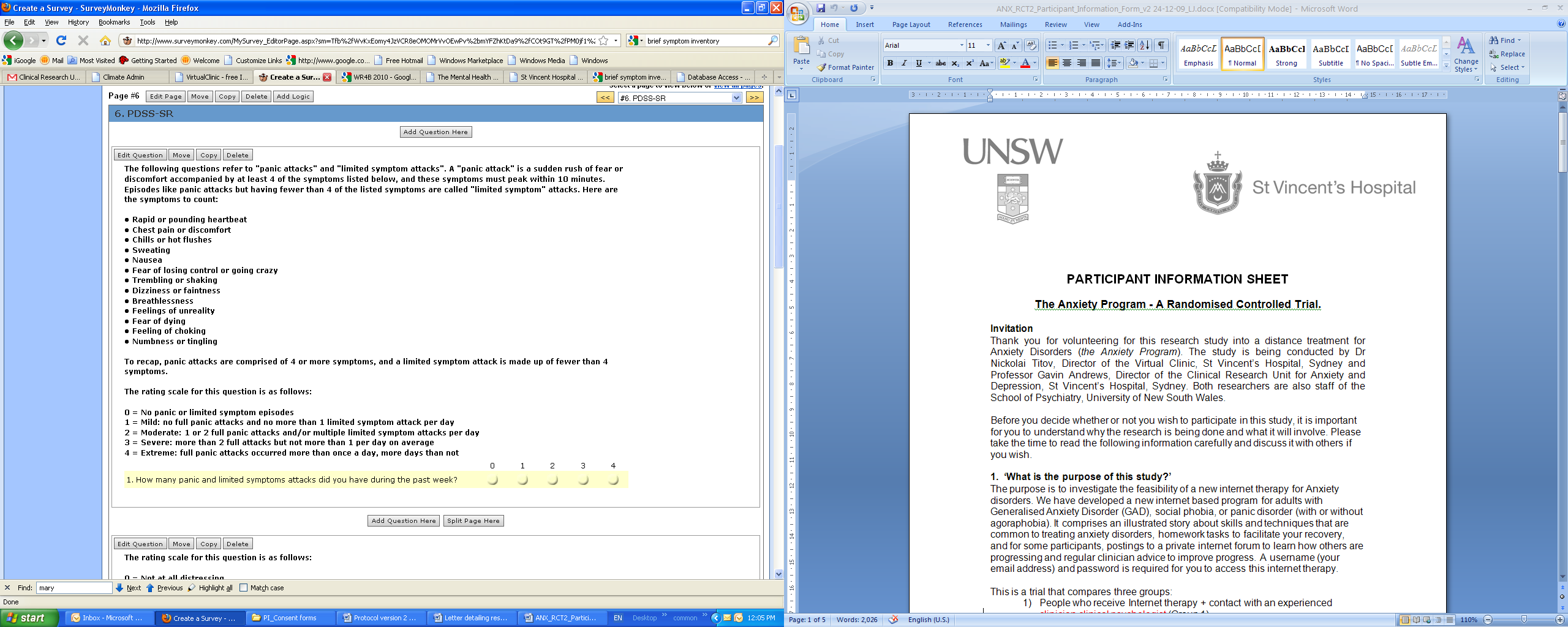


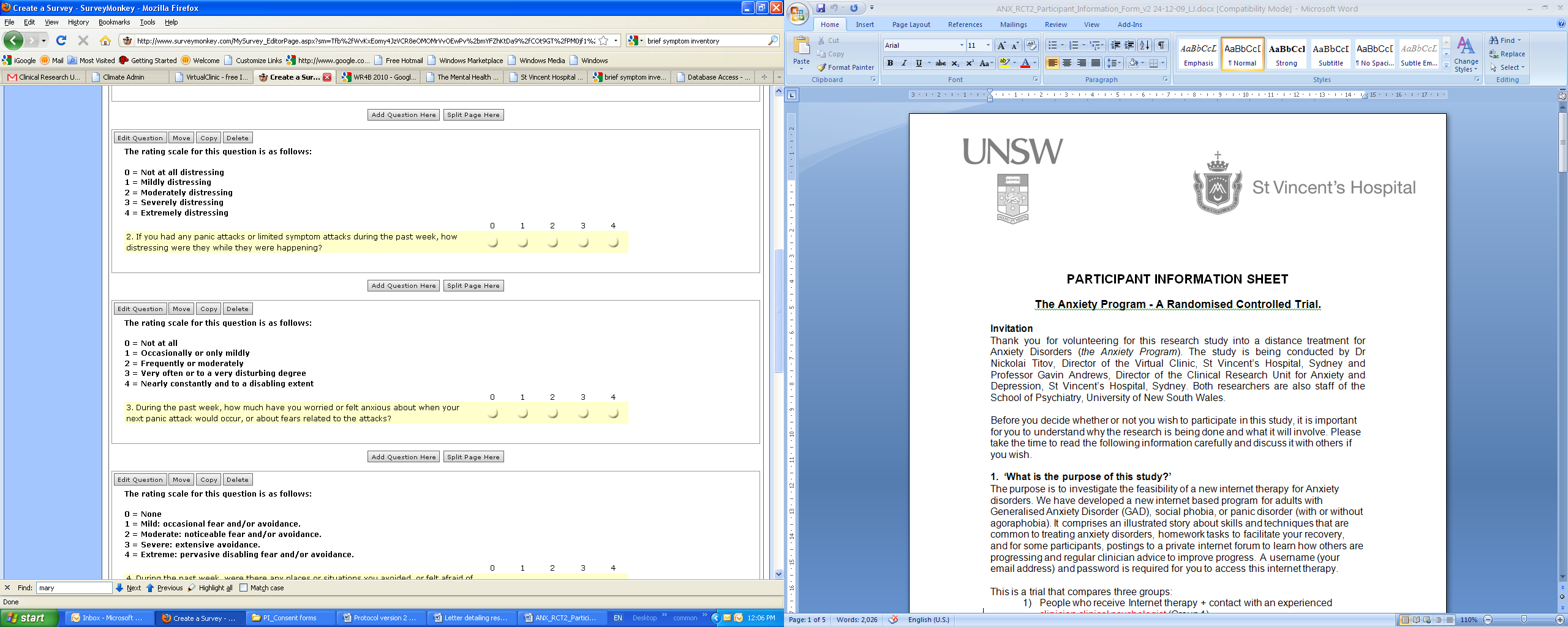


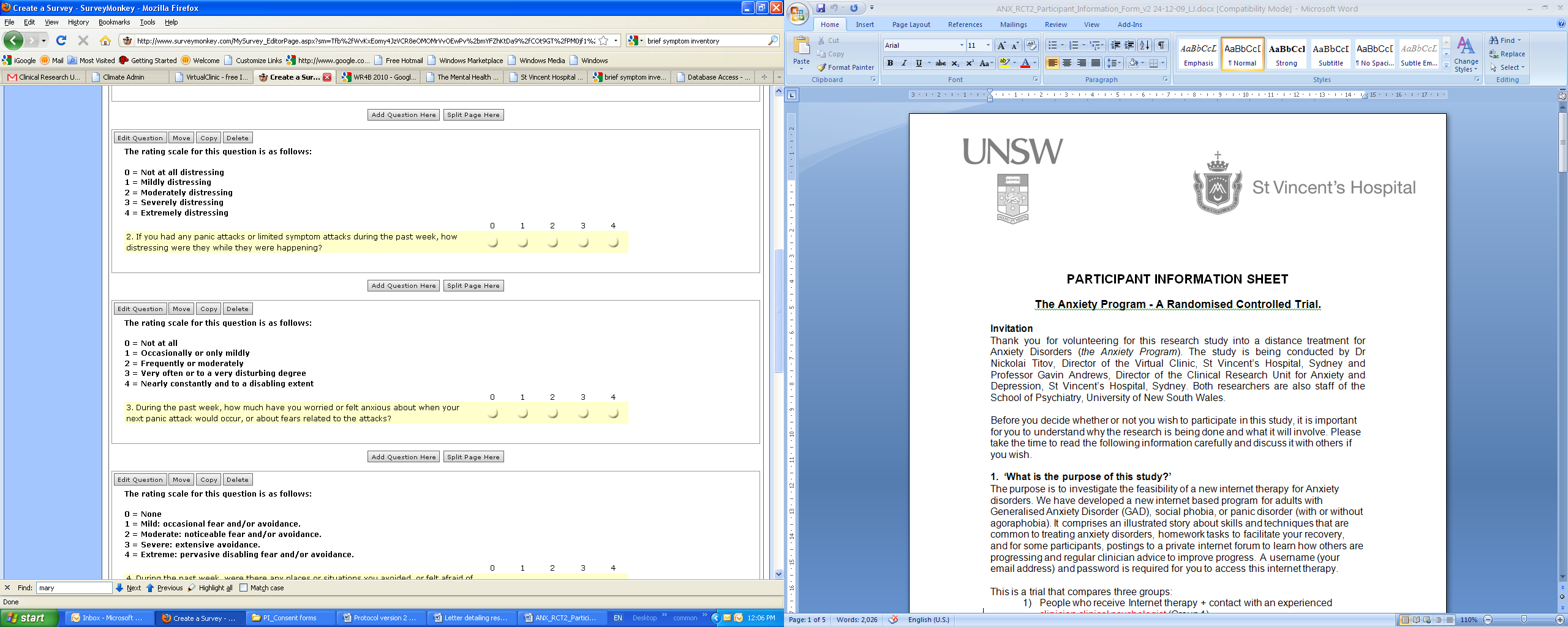


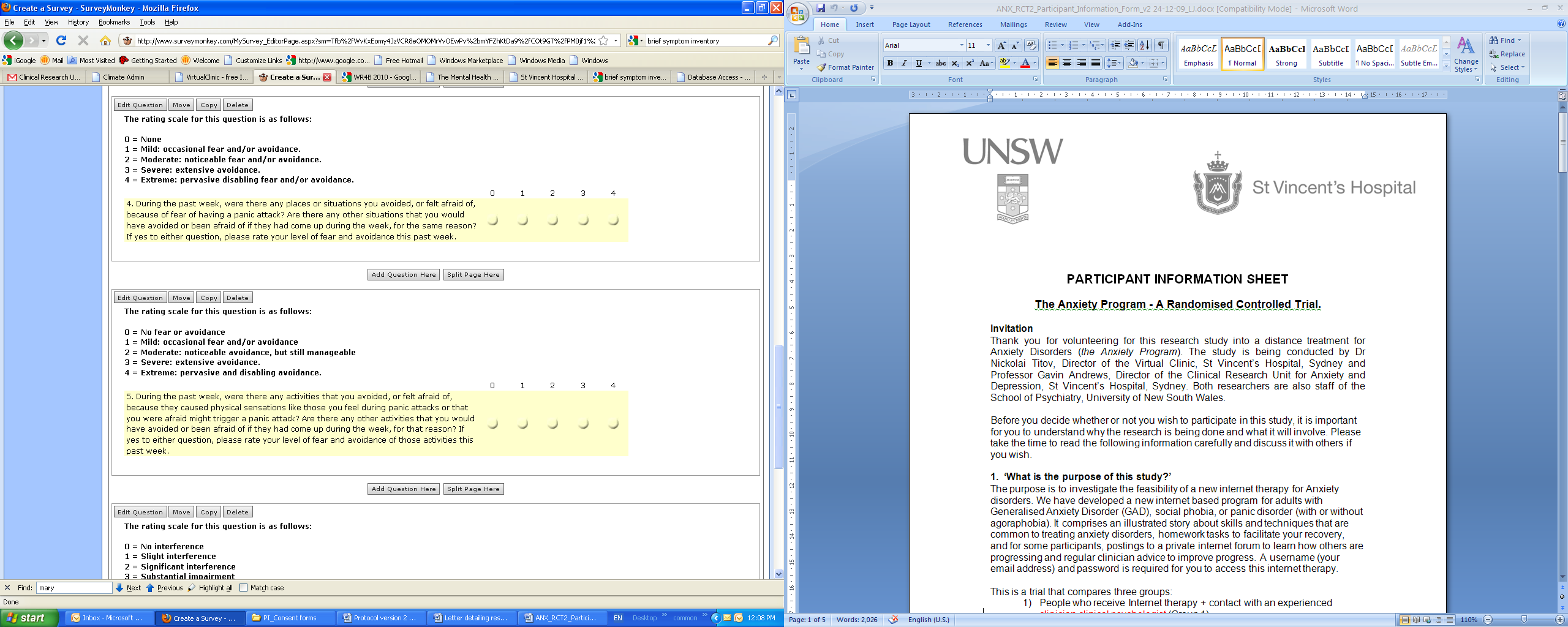


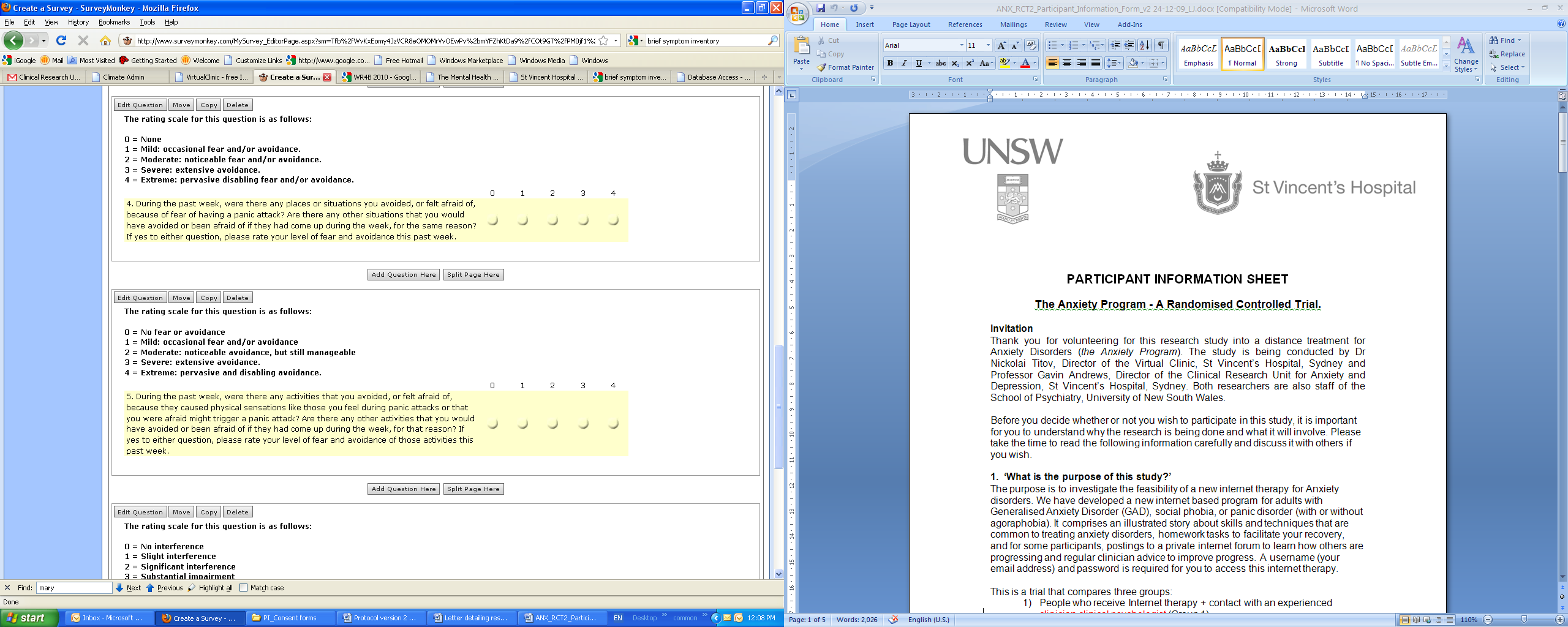


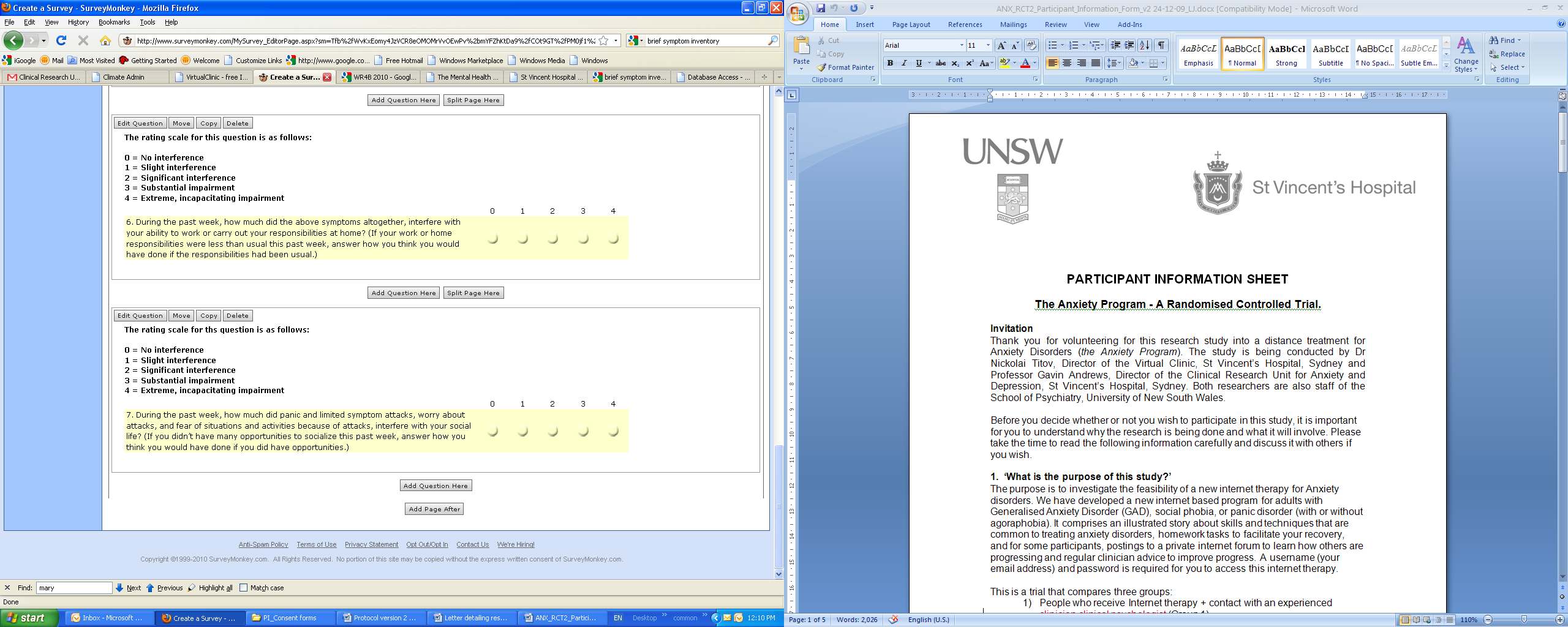


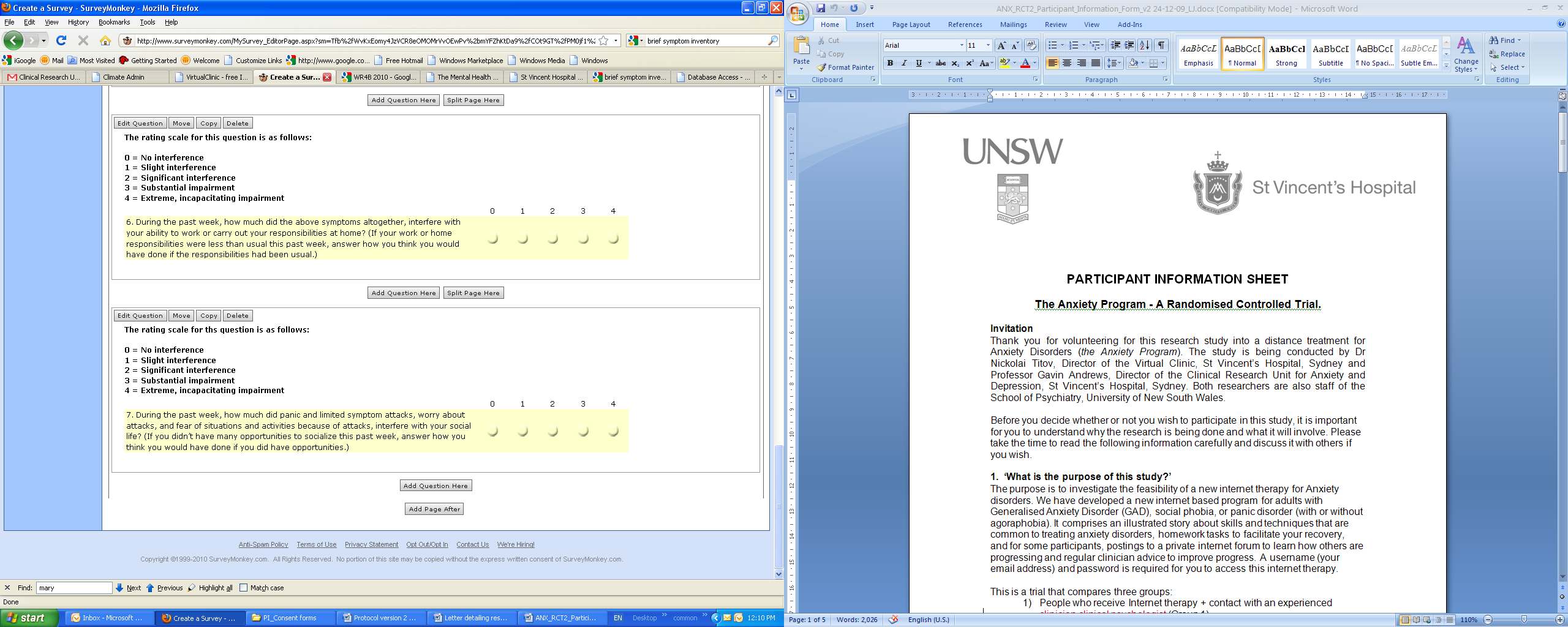


NEO-FF-I, N scale:


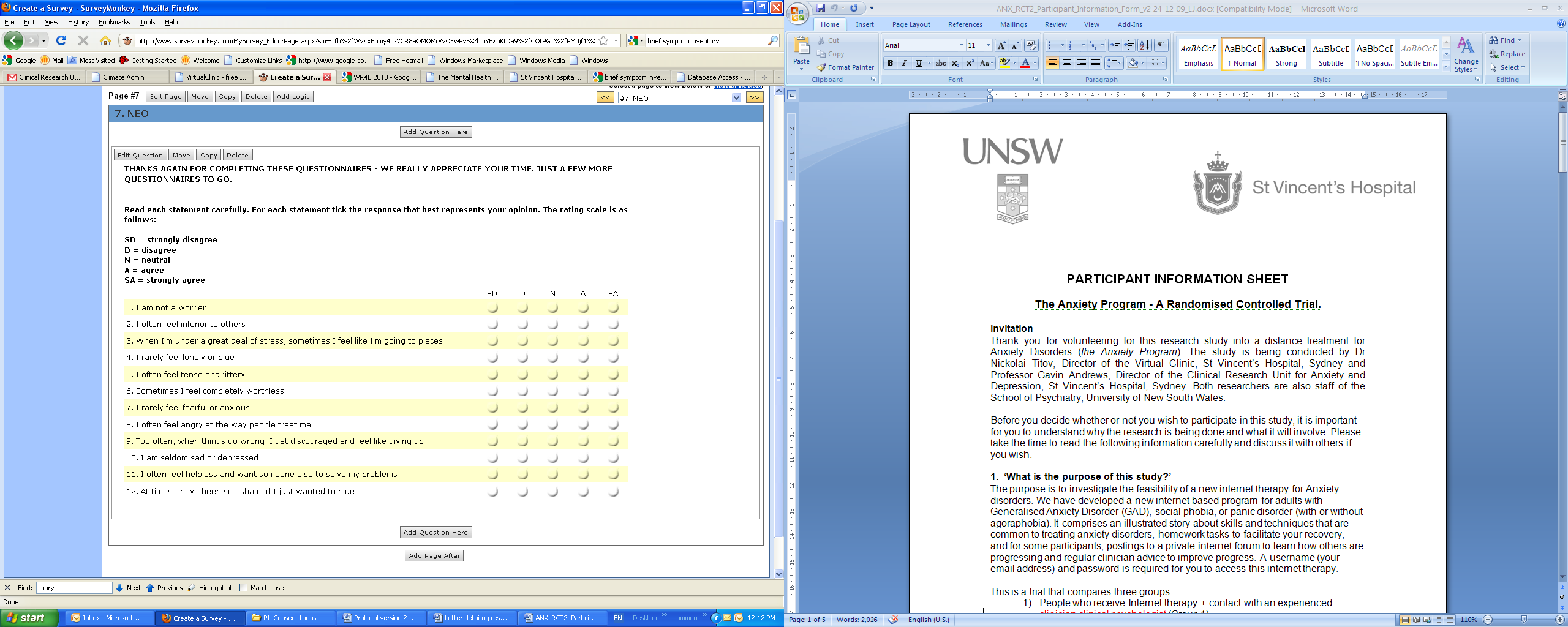


K-10:


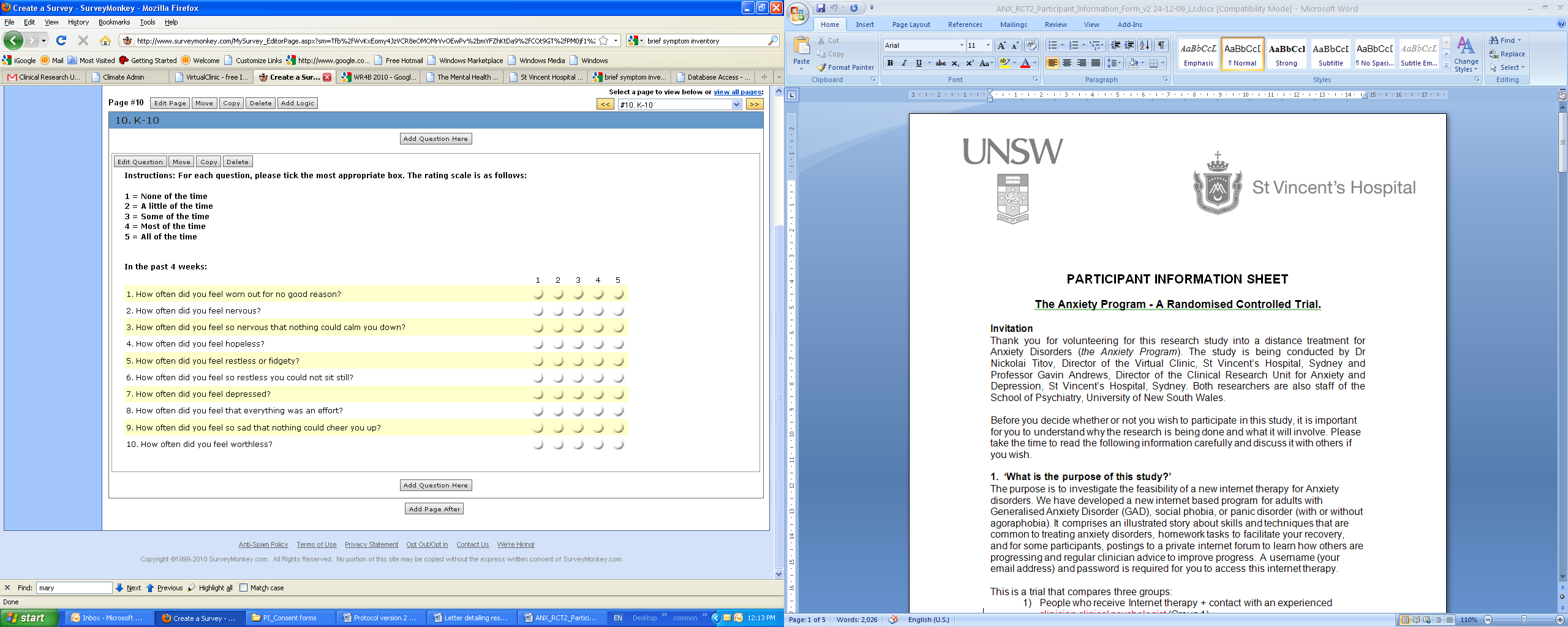


SDS:


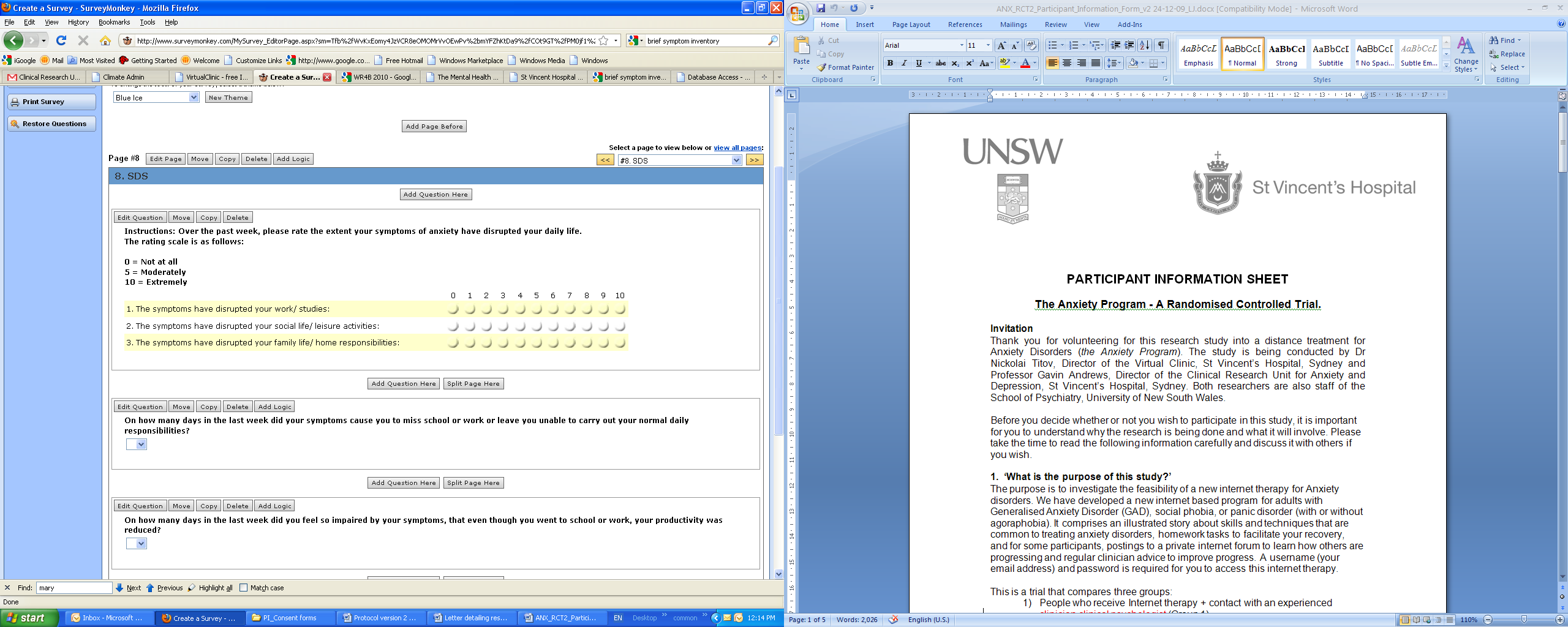


CEQ


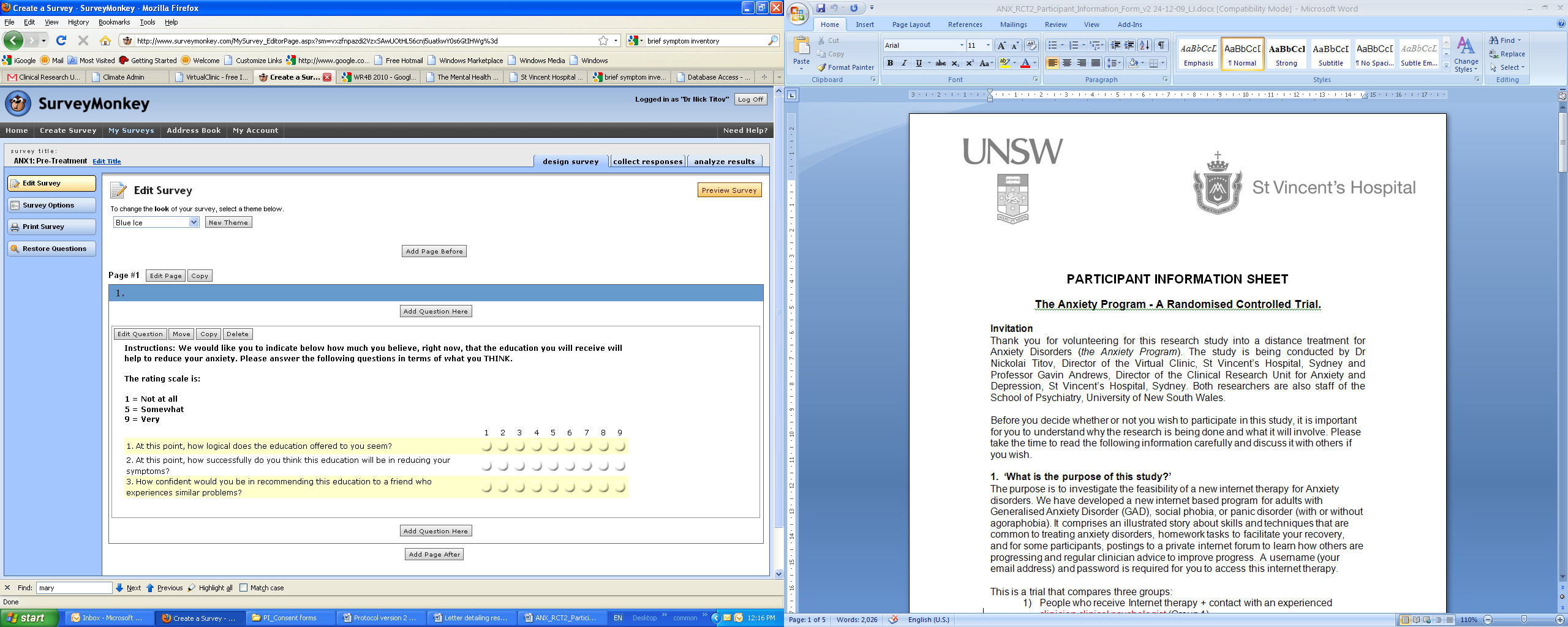


11.4 Randomisation sheet

Randomisation will occur via a true randomisation process ([www.random.org](http://www.random.org)). This list will be generated prior to commencement of the study.
